# The emerging role of circular RNAs in neurodegenerative diseases and viral infections

**DOI:** 10.1007/s13365-026-01324-8

**Published:** 2026-07-10

**Authors:** Finley Medina, Anna Bellizzi

**Affiliations:** Department of Microbiology, Immunology and Inflammation, Center for Neurovirology and Gene Editing, Lewis Katz School of Medicine, Temple University, 3500 N. Broad Street 7th floor, room 749, Philadelphia, PA 19140 USA

**Keywords:** Circular RNAs, Neurodegenerative diseases, Viral infections, Biomarkers, circRNAs therapeutics

## Abstract

Circular RNAs (circRNAs) represent a class of highly stable, covalently closed RNA molecules increasingly recognized as important regulators of brain aging and neurodegenerative disease. Growing evidence also implies circRNAs in viral infection, suggesting a potential intersection between viral neuropathogenesis and neurodegeneration. However, no studies have yet directly integrated circRNAs, neurotropic viral infections, and neurodegenerative disorders within a single mechanistic framework. To date, specific circRNAs have been linked to the progression of Alzheimer’s disease and Parkinson’s disease, where they regulate central pathological processes including amyloid-β clearance, neuroinflammation, synaptic plasticity, neuronal apoptosis, and oxidative stress. Moreover, it has been established that both host cells and viruses produce circRNAs during infection. Virus-derived circRNAs can enhance viral replication, promote immune evasion, and support latency. In contrast, host circRNAs contribute to antiviral defense by acting as microRNA sponges, interacting with viral proteins, or encoding peptides with antiviral activity, mechanisms particularly explored in viral oncogenesis. In this review, we will evaluate the most updated research evidence on the role of circRNAs in major neurodegenerative diseases and neurotropic viral infections. Considering the growing concern regarding the long-term neurological consequences of viral infections, including chronic neuroinflammation, viral reactivation, and post-viral syndromes, dysregulated circRNAs may represent a mechanistic link between viral infection and associated neurodegenerative processes. Finally, we will discuss future directions for identifying circRNAs-based biomarkers and developing circRNAs-targeted therapeutic strategies for age-related and virus-associated neurological disorders.

## Circular RNAs: a paradigm shift in RNA biology

For many years, circular RNAs (circRNAs) were considered rare byproducts of aberrant pre-mRNA splicing, regarded as transcriptional noise without functional relevance. Over the past decade, however, advances in high-throughput sequencing and dedicated bioinformatic tools have reshaped this view. CircRNAs are now recognized as abundant, conserved, and functionally significant regulatory molecules. This shift has been especially impactful in neuroscience and virology, where circRNAs have emerged as important contributors to the complex molecular networks of the nervous and immune systems. CircRNAs display remarkable structural stability, tissue- and cell-type specificity, and are particularly enriched in the brain and at synapses. (Dong et al. 2023). They are now considered a diverse and abundant class of regulatory molecules, ubiquitously expressed across eukaryotes, archaea, and even viruses (Yan and Chen 2020). Their expression often increases from embryonic to adult stages, and more than 100,000 circRNAs have been identified in the human brain alone. These characteristics, together with their detectability in body fluids such as blood and cerebrospinal fluid, make circRNAs promising diagnostic and prognostic biomarkers, especially for neurological disorders and cancer (Memczak et al. 2013; Wang et al. 2014; Amelimojarad and Amelimojarad 2025). Functionally, circRNAs are highly versatile. They can function as microRNA sponges, interact with RNA-binding proteins, modulate transcription, and in some cases encode peptides. Through these mechanisms, circRNAs influence key pathological pathways implicated in neurodegenerative diseases (NDDs), including oxidative stress, apoptosis, autophagy, mitochondrial dysfunction, and toxic protein aggregation. (Li et al. 2024b). A comprehensive understanding of circRNA biogenesis, structure, and molecular functions is essential to elucidate their emerging roles in health and disease. This review summarizes current knowledge of circRNA biology, with particular emphasis on their involvement in viral pathogenesis and neurodegeneration.

## Biogenesis and classification of circRNAs

**Fig. 1:**
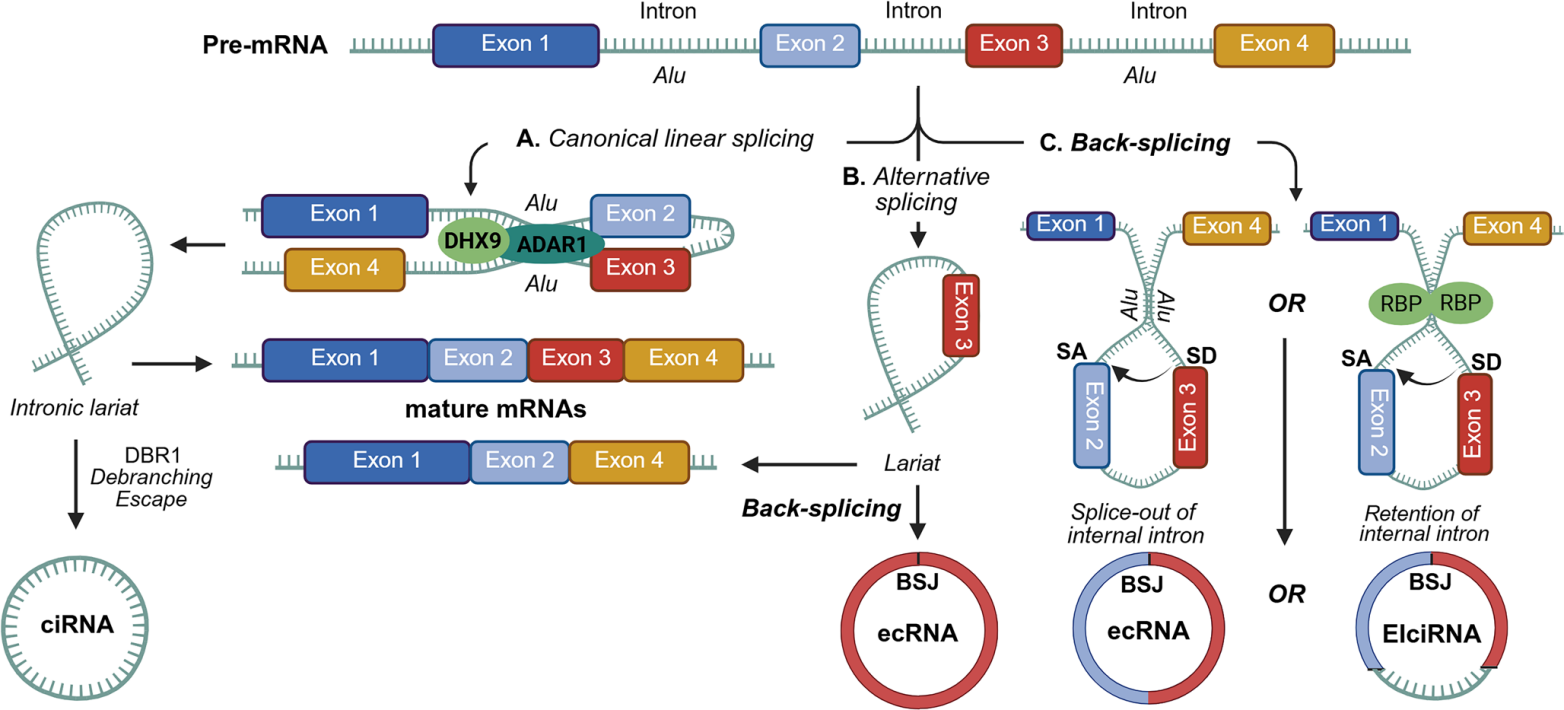
Biogenesis of circRNAs. (**A**) Circular configuration of circRNAs arises from precursor mRNAs (pre-mRNAs) through a non-canonical splicing mechanism known as back-splicing, mediated by the cellular spliceosome. Enzyme adenosine deaminase acting on RNA-1 (ADAR1), acting in combination with the androgen receptor and DExH-box helicase 9 (DHX9), can disrupt double-stranded RNA in regions of inverted repeats known as *Alu* by converting adenosine to inosine, destabilizing intronic base pairing and reducing circRNA production. The intronic lariat generated during the canonical lineal splicing can escape debranching enzyme, including debranching RNA lariats 1 (DBR1), and subsequently form circular intronic RNAs (ciRNA). (**B**) In a lariat-driven circularization model, exon skipping observed during the alternative splicing can also generate a lariat intermediate that can escape debranching enzymes and form exonic circRNAs (ecRNA). (**C**) During a competition between linear splicing and back-splicing, a downstream splice donor site (SD) can join an upstream splice acceptor site (SA), generating a closed RNA circle rather than a linear mature mRNA. This process is a tightly regulated by both *cis-acting* elements within the RNA sequence and *trans-acting* RNA-binding proteins (RBPs). Intronic complementary sequences, such as *Alu* elements, play a leading role by base-pairing across flanking. This pairing brings splice sites into proximity, favoring back-splicing over canonical splicing. RBPs proteins such as quaking (QKI) further promote circRNAs formation, by binding to motifs within flanking introns and dimerizing to bridge splice sites. During back-splicing, splice-out of internal introns can generate ecRNA, whereas retention of internal introns brings to the formation of exon–intron circRNAs (EIciRNAs). BSJ: back-splicing junction. *Created in* https://BioRender.com

CircRNAs are single-stranded RNA molecules characterized by a covalently closed loop structure that lacks the 5′ cap and 3′ poly(A) tail typical of linear messenger RNAs (mRNA). This circular configuration arises from precursor mRNAs (pre-mRNAs) through a non-canonical splicing mechanism known as back-splicing, mediated by the cellular spliceosome. During this process, a downstream splice donor site is joined to an upstream splice acceptor site, generating a closed RNA circle rather than a linear transcript. (Kristensen et al. 2019). CircRNA biogenesis is a tightly regulated event controlled by both *cis-acting* elements within the RNA sequence and *trans-acting* RNA-binding proteins (RBPs). Intronic complementary sequences, including inverted repeats such as *Alu* elements, play a central role by base-pairing across flanking introns (Fig. 1) (Zhang et al. 2014; Liang and Wilusz 2014; Ivanov et al. 2015). This pairing brings splice sites into proximity, favoring back-splicing over canonical splicing. In the lariat-driven circularization model, alternative splicing generates a lariat intermediate that can escape debranching enzymes, including debranching RNA lariats 1 (DBR1), and subsequently form a circRNA (Yan and Chen 2020; Sharma et al. 2021; Pisignano et al. 2023) (Fig. 1A and B). RBPs further modulate circRNA formation, negatively or positively regulating circularization. For example, enzyme adenosine deaminase acting on RNA-1 (ADAR1), acting in combination with the androgen receptor and DExH-box helicase 9 (DHX9), can disrupt double-stranded RNA in *Alu* regions by converting adenosine to inosine, destabilizing intronic base pairing and reducing circRNA production (Fig. 1A) (Aktaş et al. 2017; Huang et al. 2020; Erdmann et al. 2021; Shen et al. 2022). In contrast, proteins such as quaking (QKI) and muscleblind-like (MBNL) promote circRNA formation by binding to motifs within flanking introns and dimerizing to bridge splice sites (Fig. 1C) (Ashwal-Fluss et al. 2014; Conn et al. 2015; Yan and Chen 2020). The N6-methyladenosine (m6A) reader YTH domain-containing protein 1 (YTHDC1) can also enhance back-splicing of m6A-modified exons, highlighting the contribution of epi-transcriptomic modifications (Yang et al. 2017; Di Timoteo et al. 2020; Dattilo et al. 2023). Despite their high stability, circRNA levels are dynamically regulated. Certain endonucleases can cleave specific internal motifs: for example, argonaute-2 (AGO2) can mediate circRNA degradation through a microRNA (miR)-guide, in a RNA interference-like manner (Pan et al. 2019; Amelimojarad and Amelimojarad 2025). Additional RBPs, such as up-frameshift protein 1 (UPF1), may bind and unwind circRNAs to facilitate enzymatic degradation (Fischer et al. 2020).

CircRNAs are classified into three main categories: exonic circRNAs (ecRNAs), circular intronic RNAs (ciRNAs), and exon–intron circRNAs (EIciRNAs) (Zhang et al. 2020c; Titze-de-Almeida and Titze-de-Almeida 2023). EcRNAs, composed solely of exons, represent more than 80% of circRNAs and are predominantly cytoplasmic (Fig. 1B and C) (Meng et al. 2017). CiRNAs derive exclusively from introns and remain mainly in the nucleus, where specific guanine/uracil-rich and cytosine-rich sequence elements prevent debranching (Fig. 1A) (Zhang et al. 2013). EIciRNAs retain both exons and introns and are also enriched in the nucleus, where often are involved in transcriptional regulation (Fig. 1C) (Shen et al. 2015; Chen 2016). The circular topology of circRNAs confers resistance to exonucleases, resulting in half-lives at least 2.5 times longer than those of linear mRNAs **(**Kristensen et al. 2019). This enhanced stability is particularly advantageous in long-lived cells such as neurons. CircRNAs are often expressed at high levels, display tissue specificity, and are evolutionarily conserved. They are especially abundant in the brain, including the cortex, hippocampus, and white matter, where their expression increases with age and is dynamically regulated during neuronal differentiation and synaptic activity (Huang et al. 2020). Functionally, circRNAs participate in epigenetic, transcriptional, and post-transcriptional regulation and are implicated in multiple neurological disorders (Dong et al. 2023). Their remarkable stability and detectability in biofluids such as blood and cerebrospinal fluid make them promising minimally invasive biomarkers for the diagnosis of NDDs and viral infection (Li et al. 2024b).

## Functional repertoire of cellular circRNAs

A substantial and expanding body of evidence demonstrates that a subset of circRNAs performs distinct and physiologically meaningful functions. Their evolutionary conservation, cell-type-specific expression, and dynamic regulation independent of their linear parental transcripts strongly support active biological roles. CircRNAs participate in gene regulation through four main mechanisms: (1) they can function as microRNA (miRNA) sponges; (2) interact with RBPs as decoys, scaffolds, or recruiters; (3) serve as templates for protein translation; (4) and modulate transcription of their parental genes (Kristensen et al. 2019; Yan and Chen 2020).

**Fig. 2:**
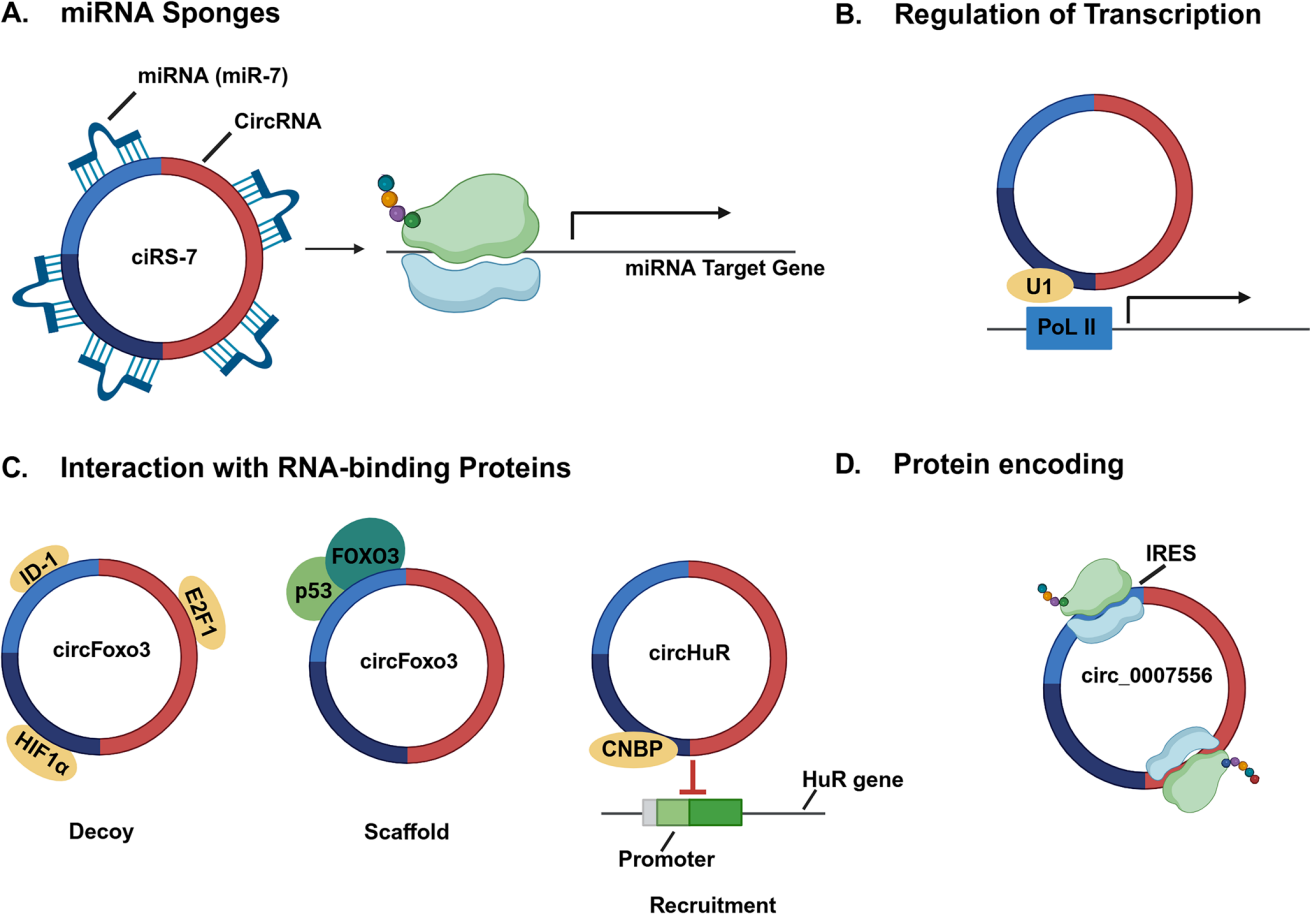
Functional repertoire of cellular circRNAs. (**A**) Circular RNAs (CircRNAs) contain binding sites for microRNAs (miRNAs) sponging, which prevents miRNAs from binding to their target messenger RNAs (mRNAs) and thus regulating gene expression. The prototypical example of this function is ciRS-7 (or CDR1as), which harbors more than 70 binding sites for miR-7 and potently inhibits its own activity or UBE2A and α-synuclein expression, linking circRNA activity to neurodegenerative pathways. (**B**) CircRNAs can interact with the transcriptional machinery to regulate the expression of their parental genes. Intron-containing circRNAs predominantly nuclear, can associate with U1 small nuclear ribonucleoproteins (snRNPs) and RNA polymerase II (Pol II) to enhance transcription of their parental genes. (**C**) CircRNAs can bind to proteins to alter their function, stability, or cellular location, acting as protein decoys, scaffolds, or recruiters. CircFoxo3 can act RNA-binding proteins (RPBs) decoys, binding inhibitor of DNA binding 1 (ID-1), E2F transcription factor 1 (E2F1), and hypoxia-inducible factor-1 alpha (HIF-1α) and limiting their nuclear entry. CircFoxo3 can also scaffold interactions between Foxo3 and p53. circHuR, as recruiter circRNA, can interact with the protein CNBP to inhibit the binding of CNBP to the HuR promoter, thereby downregulating HuR expression. (**D**) While most are non-coding, some circRNAs contain internal ribosome entry sites (IRES), which are structured RNA sequences capable of directly recruiting the ribosome to an internal site on the RNA to initiate translation. An example of protein-coding circRNAs includes circ_0007556, reported to encode an amyloid-β (Aβ)-related peptide. Created in https://BioRender.com

One of the earliest and best-characterized functions of circRNAs is their role as miRNA sponges within the competitive endogenous RNA (ceRNA) network (Fig. 2A). CircRNAs containing multiple miRNA response elements (MREs) can sequester specific miRNAs, thereby relieving repression of target mRNAs (Memczak et al. 2013). The prototypical example of this function is CDR1as/ciRS-7, which harbors more than 70 binding sites for miR-7 and potently inhibits its own activity. In neurological contexts, ciRS-7-mediated sequestration of miR-7 influences targets such as UBE2A and α-synuclein, linking circRNA activity to neurodegenerative pathways (Zhao et al. 2016). Other circRNAs, including circGFRA1 (circ_005239) and circ_0000950, regulate disease-relevant processes, like proliferative pathways and downstream inflammatory signaling, by modulating miRNA availability (miR-99a and miR-103 respectively) (Yang et al. 2019b; Cao et al. 2021; He et al. 2022; Li et al. 2024b). However, genome-wide analyses indicate that most circRNAs do not contain unusually high densities of miRNA binding sites, suggesting that miRNA sponging is a specialized function limited to specific circRNAs rather than a universal property (Yan and Chen 2020). Because back-splicing competes with canonical splicing, circRNA formation can directly influence the output of linear mRNA from the same gene. EIciRNAs, predominantly nuclear, can associate with U1 small nuclear ribonucleoproteins (snRNPs) and RNA polymerase II (Pol II) to enhance transcription of their parental genes (Fig. 2B). For example, circEIF3J and circPAIP2 promote transcription of their parental genes through such interactions (Bentley 2014; Chen et al. 2015; Qu et al. 2015; Zhang et al. 2020c; Li et al. 2024b). The circMbl locus in *Drosophila* illustrates a feedback mechanism: the MBL protein enhances circularization of its own transcript to form circMbl. Increased circMbl binds and sequesters MBL protein reducing linear Mbl mRNA output and creating a negative feedback loop that fine-tunes protein levels (Ashwal-Fluss et al. 2014; Bose and Ain 2018). Some circRNAs also regulate gene expression by altering DNA methylation. CircACR interacts with DNA methyltransferase 3β to reduce methylation at the Pink1 promoter, thereby activating its expression (Zhou et al. 2019; Titze-de-Almeida and Titze-de-Almeida 2023).

Direct interactions with RBPs represent another major functional category. CircRNAs can bind proteins to modulate their localization, stability, or activity, or serve as scaffolds assembling multi-protein complexes (Fig. 2C). CircDNMT1 promotes nuclear translocation of p53 and AU-rich element-binding factor 1 (AUF1) in breast cancer (Du et al. 2018). CircFoxo3 binds inhibitor of DNA binding 1 (ID-1), E2F Transcription Factor 1 (E2F1), and hypoxia-inducible factor-1 alpha (HIF-1α), limiting their nuclear entry, and can scaffold interactions between Foxo3 and p53 (Du et al. 2016, 2017; Huang et al. 2020). CircPABPN1 sequesters human antigen R (HuR), inhibiting its function, whereas circSKA3 binds integrin β1 to enhance invasive behavior in cancer cells (Abdelmohsen et al. 2017; Du et al. 2020; Li et al. 2024b). In the nervous system, circGRIA1 modulates synaptic plasticity by interacting with glutamate receptor 1, and circHuR interacts with the protein CNBP to inhibit the binding of CNBP to the HuR promoter, thereby downregulating HuR expression (Yang et al. 2019a, 2020; Xiao et al. 2024).

Although initially classified as non-coding, certain circRNAs are now known to encode proteins or microproteins (< 100 amino acids). Because they lack a 5′ cap, their translation depends on cap-independent mechanisms such as internal ribosome entry sites (IRES), which are structured RNA sequences capable of directly recruiting the ribosome to an internal site on the RNA to initiate translation, or N6-methyladenosine (m6A)-mediated initiation. m6A-driven translation requires factors including eukaryotic translation initiation factor 4 gamma 2 (eIF4G2) and the m6A “reader” protein (YTHDF3) to recruit ribosomes (Kristensen et al. 2019; Yan and Chen 2020; Sharma et al. 2021) (Fig. 2D). Examples of protein-coding circRNAs include (1) circZNF609, which encodes a protein involved in myogenesis (Legnini et al. 2017); (2) circGFP, a synthetic circRNA that translates a fully functioning GFP protein *in vitro* (Wang and Wang 2015); (3) circFBXW7, which produces a tumor-suppressive microprotein (Yang et al. 2018b); and circ_0007556, reported to encode an amyloid-β (Aβ)-related peptide (Mo et al. 2020). Collectively, these diverse mechanisms underscore the multifaceted roles of circRNAs in regulating gene expression and cellular function across physiological and pathological contexts.

## CircRNAs in neurodegenerative disease

Neurodegenerative diseases (NDDs) constitute a major and growing global health challenge, particularly in aging populations. These disorders are characterized by progressive neuronal dysfunction and loss in the central and peripheral nervous systems, typically manifesting between 50 and 70 years of age (Xiao et al. 2024). NDDs can be classified according to clinical presentation, anatomical distribution of neurodegeneration, or the predominant misfolded protein species. Broadly, they include acute conditions, such as cerebral ischemia, traumatic brain injury, and epilepsy, and chronic disorders, including Alzheimer’s disease (AD), Parkinson’s disease (PD), amyotrophic lateral sclerosis (ALS), Huntington’s disease (HD) and spinocerebellar ataxia (SCA) (Xiao et al. 2024). Despite advances in neurobiology, current diagnostic approaches remain limited, often relying on non-specific clinical manifestations or invasive biomarker assessments, such as Aβ and α-synuclein (α-syn), which typically detect disease only after substantial neuronal damage has occurred (Li et al. 2024b). Nowadays, circular RNAs (circRNAs) have emerged as promising candidates for improving both diagnosis and mechanistic understanding of NDDs. Distinct circRNA expression profiles have been identified across multiple neurodegenerative conditions. In chronic disorders such as AD, PD, and ALS, specific circRNAs are consistently up- or down-regulated, correlating with disease status and highlighting their potential as biomarkers and therapeutic targets (Li et al. 2024b). Recent transcriptomic analyses have revealed striking cell-type specificity in circRNA expression within the human brain (Dong et al. 2023). Thousands of circRNAs show preferential enrichment in dopamine neurons, pyramidal neurons, or non-neuronal cells, suggesting highly specialized regulatory functions. Notably, genetic risk loci associated with addiction preferentially express circRNAs in dopamine neurons, whereas loci linked to autism and bipolar disorder show enrichment in pyramidal neurons. In contrast, oncologic diseases such as leukemia are associated with circRNAs predominantly expressed in non-neuronal cells. Importantly, the linear mRNAs transcribed from the same loci often lack comparable cell-type specificity, indicating that circRNAs may provide critical regulatory signatures that shape neuronal identity and vulnerability (Dong et al. 2023). Alternative back-splicing from “super-host” loci further enhances this molecular diversity. For example, the ERC1 locus generates distinct circRNA isoforms enriched in different neuronal populations, while its linear transcript displays a separate expression pattern. ERC1 binds to the Parkinson’s Disease (PD)-linked Rab-interacting molecule (RIM) proteins to facilitate the docking of synaptic vesicles at the presynaptic active zone (Nalls et al. 2019; Liu et al. 2021). Such findings suggest that circRNAs are not passive byproducts but active regulators of neuronal function (Dong et al. 2023). Cell type-specific dysregulation of circERC1 and other circRNAs, including circDNAJC6 in early PD (Elsayed et al. 2016; Olgiati et al. 2016), supports their pivotal involvement in synaptic dysfunction and neurodegeneration. This review summarizes the most extensively studied dysregulated circRNAs in AD, PD, and ALS, and discusses their potential contributions to the molecular pathways underlying chronic NDDs.

### circRNAs in Alzheimer’s disease pathogenesis

Alzheimer’s disease (AD) is a chronic, progressive neurodegenerative disorder and the leading cause of dementia worldwide, particularly among older adults (Pierouli et al. 2022; Alzheimer’s Association 2025**)**. Its development reflects a complex interplay of genetic susceptibility, environmental exposures, and lifestyle-related factors. Although most cases are sporadic, the small proportion of familial AD cases (approximately 1–5%) arise from autosomal dominant mutations in genes directly involved in amyloid precursor protein (APP) processing, including APP itself and the presenilin genes PSEN1 and PSEN2. These mutations alter γ-secretase activity and promote overproduction of amyloid-β (Aβ) peptides, particularly the aggregation-prone Aβ42 species (Bateman et al. 2010; Lane et al. 2018). In contrast, the far more common sporadic form of AD (> 95% of cases) lacks these deterministic mutations, but it is strongly influenced by genetic risk factors, most notably the apolipoprotein E (APOE) ε4 allele. Carrying one APOE4 allele significantly increases AD risk, while homozygosity confers an approximately ten-fold elevation in susceptibility. ApoE4 protein is less efficient in lipid transport and Aβ clearance, and poorly lipidated ApoE4 can bind Aβ42 in ways that favor aggregation and impair removal from the brain (Saunders et al. 1993; Millan 2017; Krokidis et al. 2021). Clinically, AD manifests as a gradual and relentless decline in cognitive abilities. Early symptoms typically involve episodic memory impairment, followed by deficits in executive function, language, visuospatial abilities, and eventually profound global cognitive deterioration accompanied by behavioral and personality changes. Neuropathologically, the disease is characterized by selective vulnerability of the hippocampus, entorhinal cortex, neocortex, and amygdala. Three principal hallmarks define AD pathology: extracellular amyloid plaques composed of aggregated Aβ peptides, intracellular neurofibrillary tangles (NFTs) formed by hyperphosphorylated tau protein, and widespread neuronal and synaptic loss (Lane et al. 2018; Jia et al. 2021; Beylerli et al. 2024). Aβ peptides are generated through sequential cleavage of APP by β-secretase (BACE1) and γ-secretase. In familial AD, mutations increase Aβ production or shift the ratio toward the more toxic Aβ42 form. However, in sporadic AD, the precise origin of excess Aβ remains incompletely understood. While elevated BACE1 activity has been reported in some sporadic cases, total APP expression and γ-secretase activity are often comparable to controls (Chouraki and Seshadri 2014; Aubry et al. 2015; Greenough 2016; Huang et al. 2017). Intriguingly, transgenic mouse models overexpressing wild-type human APP, constructed in ways that preclude circular RNA formation, do not always develop robust plaque pathology, suggesting that alternative transcripts or regulatory pathways may contribute to Aβ generation (Mo et al. 2020). NFTs represent the second major pathological feature. Tau is a microtubule-associated protein essential for stabilizing the neuronal cytoskeleton and supporting axonal transport. In AD, tau becomes abnormally hyperphosphorylated due to imbalances between kinase and phosphatase activity. Hyperphosphorylated tau detaches from microtubules, misfolds, and aggregates into insoluble filaments that disrupt intracellular transport and synaptic integrity. The combined toxicity of Aβ accumulation, tau pathology, oxidative stress, and chronic neuroinflammation culminates in progressive synaptic failure and neuronal death (Lane et al. 2018).

In recent years, circRNAs have emerged as important regulators in the molecular landscape of AD (Huang et al. 2020). Their expression increases during neuronal differentiation and synaptogenesis, and their abundance is often independent of the linear transcripts produced from the same gene locus, suggesting distinct biological functions (Bigarré et al. 2021). Large-scale transcriptomic studies have demonstrated that circRNAs expression profiles are significantly altered in AD (Dube et al. 2019). One comprehensive analysis of human brain tissues identified dozens of circRNAs associated with clinical dementia rating, Braak score, and AD diagnosis. Notably, some circRNAs showed more variance in cognitive measures than APOE4 allele levels, highlighting their potential functional importance (Dube et al. 2019). An additional study on differential expressions of circRNAs in middle temporal (MT) cortex of a well-characterized cohort of human AD brain tissues, identified specific circRNAs linked to neuropathological burden (Bigarré et al. 2021). Specifically, circ_0131235, generated from insulin-like growth factor-2 receptor (IGF2R) gene, has been found significantly upregulated in the MT cortex of individuals with advanced Braak stage pathology. Interestingly, its expression correlates with neuropathological severity rather than cognitive impairment per se, suggesting it may serve as a biomarker of underlying pathology (Bigarré et al. 2021). The parental gene, IGF2R, participates in Aβ clearance pathways, raising the possibility that increased circRNA expression represents a compensatory response to amyloid accumulation (Table 1) (Bigarré et al. 2021).

**Table 1:** AD-related circRNAs

Gene	Trend	circRNA Function	Target gene and Functional Pathway	Source	Functional activity	References
circ_0000950	Up	miRNA sponges	circ_0000950↑ → miR-103↓ → PTGS2↑ → neurite outgrowth↓, inflammatory cytokines (IL-1β, IL-6, TNF-α) ↑ → neuron apoptosis↑	Cellular AD model of rat PC12 cells and cerebral cortical neurons.	Involved in neuroinflammation	(Yang et al. 2019b)
ciRS-7 (or CDR1as)	Down	miRNA sponges	ciRs-7↓ → miR-7↑ → UBE2A↓ → Aβ and senile plaque deposits↑	Brain tissues of AD patients	Regulates amyloid peptide clearance	(Zhao et al. 2016)
ciRS-7 (or CDR1as)	Up	Regulation of transcription	ciRs-7↑ → NF-κB↓ → UCHL1↑ →APP and BACE1↓ → Aβ↓	Cellular AD model	Regulates amyloid peptide clearance	(Shi et al. 2017)
circHDAC9 (circ_0003594)	Down	miRNA sponges	circHDAC9↓ → miR-138↑ → SIRT1↓ → Aβ↑	Sera of MCI and AD patients. Hippocampus of AD mouse model	Leads to synaptic/memory deficits	(Lu et al. 2019)
circHDAC9 (circ_0003594)	Up	miRNA sponges	Barberine → circHDAC9↑ → miR-142-5p↓→ Aβ↓	Cellular AD model	Alleviates Aβ42 formation	(Zhang et al. 2020b)
circCwc27	Up	Decoying RNA-Binding Protein	circCwc27↑ → Pur-α’s retention in the cytoplasm → Aβ deposition↑	Cortex and hippocampus of AD mouse model and AD patients’ brain	Dysregulated Pur-α, and enhancer of AD-associated genes’ promoter.	(Song et al. 2022)
circAβ-a (circ_0007556)	Up	Protein Encoding	circAβ-a↑ → Aβ↑	AD human entorhinal cortex and cellular AD model	Cleaved into Aβ-peptides, plays a role in sporadic AD pathogenesis	(Mo et al. 2020; Urdánoz-Casado et al. 2023)
circ_0004381	Up	miRNA sponges	circ_0004381↑ → miR-647↓ → PSEN1↑ → Aβ↑	AD mouse model	Induces neuronal damage and neuroinflammation.	(Li et al. 2022a)
circ_0131235 (circIGF2R)	Up	Unknown	circ_0131235↑ → IGF2R dysregulation	Middle temporal cortex of AD patients	AD biomarker and molecular link between metabolic dysfunction and AD	(Bigarré et al. 2021)
circLPAR1 (circ_0003611)	Up	miRNA sponges	circLPAR1↑ → mir-212-3p↓ → ZNF217↑	CSF of AD affected people	Speeds up apoptosis, inflammation, and oxidative stress* in vitro*	(Li et al. 2020b; Wu et al. 2021)
circLPAR1 (circ_0003611)	Up	miRNA sponges	circ_0003611↑ → miR-383-5p↓ → KIF1B↑	SH-SY5Y and SK-N-SH cells treated with Aβ	Enhancer of Aβ-mediated apoptosis, inflammation and oxidative stress.	(Li et al. 2022c).
circLPAR1 (Circ_0003611)	Up	miRNA sponges	circ_0003611↑ → miR-885-5p↓ → Kremen1↑ → Aβ-induced neuronal injury↑	Aβ-treated SK-N-SH cells	Enhancer of Aβ-induced neuronal cell injury	(Pan et al. 2022)
circGPHN (circ_0032253)	Up	Regulation of Transcription	circGPHN↑ → GPHN↓	CSF of AD affected people	Involved in the integrity of the inhibitory synapse.	(Smolinsky et al. 2008; Hales et al. 2013; Li et al. 2020b)
circAXL (circ_0002945)	Up	Regulation of Transcription	circAXL ↑ → AXL↓	CSF of AD affected people	Dampens the inflammation and reduces the clearance of apoptotic neuronal cells.	(Weinger et al. 2011; Li et al. 2020b)
circAXL (circ_0002945)	Up	miRNA sponges	AXL↑ → miR-328↓ → BACE1↑ → neuron injury↑	SH-SY5Y and SK-N-SH cells treated with Aβ	Increases neuron injury and inflammation	(Li et al. 2022b)
circPCCA (circ_003077)	Down	miRNA sponges	circ-PCCA↑ → 138-5p↓ → tau phosphorylation↓	CSF of AD affected people	Inhibits tau phosphorylation	(Wang et al. 2015; Li et al. 2020b)
circKIAA1586	Up	miRNA sponges	circRNA KIAA1586↑ → miR-29b↓, miR-101↓, miR-15a↓→ BACE↑ and APP↑ → Aβ↑	In silico expression profiles of AD-related circRNA-associated ceRNA network.	Dysregulates the expression of BACE1 and/or APP, influencing Aβ pathology	(Hébert et al. 2008; Long and Lahiri 2011; Pereira et al. 2016; Zhang et al. 2019)
circHOMER1	Down	miRNA sponges	circHOMER1↓ → miR-651↑ → PSEN1/PSEN2↓ → Aβ↓	Olfactory and frontal cortex of AD brains	Novel marker of AD risk and diagnosis	(Dube et al. 2019; Cervera-Carles et al. 2020; Urdánoz-Casado et al. 2021)
circCORO1C	Up	miRNA sponges	circCORO1C↑ → miR-105↓ → SNCA↓	Cortex of AD brains	Reduced accumulation of Aβ and α-syn in neurons	(Dube et al. 2019)
circPIK3C3 (circ_000843)	Up	Unknown	Unknown	PBMC of AD patients	PIK3C3 is involved in regulation of APP processing	(Li et al. 2020c)
circUBASH3B (circ_0003391)	Down	miRNA sponges	circ_0003391↓ → miR-574-5p↑ in AD patients	Peripheral blood of AD patients	Potential AD biomarker	(Liu et al. 2020)
circPTK2 (circ_0008305)	Up	miRNA sponges	circPTK2↑ → miR-181c-5p↓ → HMGB1↑	Microglia of AD mouse model	Modulates microglial activity and neuroinflammation	(Li et al. 2021 a)

*Abbreviation.* *Up* up-regulated, *Down* down-regulated, *miRNA* microRNA, *PTGS2 *prostaglandin endoperoxide synthase 2,* IL* interleukin, *TNF* tumor necrosis factor, *AD *Alzheimer’s disease, *UBE2A* ubiquitin-conjugating enzyme 2 A, *Aβ* amyloid beta peptide, *NF-κB* nuclear factor kappa B, *UCHL1* ubiquitin carboxyl-terminal hydrolase L1, *APP* amyloid precursor protein, *BACE1 *beta-site APP Cleaving enzyme 1 or β-secretase, *MCI *mild cognitive impairment, *SIRT1 *sirtuin 1, *PSEN1 and PSEN2* presenilin-1 and − 2, *IGF2R* insulin-like growth factor-2 receptor, *ZNF217* zinc finger protein 217, *CSF* cerebrospinal fluid, *KIF1B* kinesin family member 1B, *GPHN* gephyrin gene, *AXL* anexelekto, member of the TAM (TYRO3, AXL, MERTK) family of receptor tyrosine kinases, *ceRNA* competitive endogenous RNA, *SNCA* α-synuclein gene, *PBMC* peripheral blood mononuclear cells, *PIK3C3 *phosphatidylinositol 3-kinase catalytic subunit type 3, *HMGB1* high mobility group protein B1

CircRNAs influence AD pathogenesis through multiple mechanisms. A central pathway involves modulation of Aβ homeostasis. The circRNA ciRS-7 (CDR1as), a well-characterized sponge for miR-7, is downregulated in the hippocampus of sporadic AD patients. Reduced ciRS-7 permits increased miR-7 activity, which suppresses ubiquitin-conjugating enzyme UBE2A, a key component of proteasomal Aβ clearance. Impaired proteostasis contributes to amyloid accumulation (Zhao et al. 2016). Conversely, ciRS-7 has also been reported to influence APP and BACE1 degradation through NF-κB-dependent mechanisms by upregulating ubiquitin carboxyl-terminal hydrolase L1 (UCHL1) transcription, underscoring its complex regulatory role (Table 1) (Shi et al. 2017; Zhang et al. 2020c). circHDAC9 is another extensively studied circRNA that regulates APP processing via miRNA-mediated networks. It sponges miR-138, thereby promoting expression of sirtuin 1 (SIRT1), a protein associated with reduced Aβ accumulation and improved mitochondrial function. In AD models, circHDAC9 is downregulated, leading to elevated miR-138, suppression of the non-amyloidogenic α-secretase (ADAM10) pathway, and increased Aβ42 production. Importantly, reduced circHDAC9 levels have been detected in serum from patients with mild cognitive impairment and AD, highlighting its biomarker potential (Lu et al. 2019). CircHDAC9 also binds to miR-142-5p upregulated by Aβ, and overexpression of circHDAC9 alleviates Aβ-induced neurotoxicity, including reduced cell viability, apoptosis, and inflammation. Berberine treatment was found to have a protective effect against Aβ-induced neurotoxicity, partly by regulating the circHDAC9/miR-142-5p axis and increasing circHDAC9 expression (Zhang et al. 2020b). circKIAA1586 can also sponge miR-29b, miR-101, and miR-15a, which target and reduce the expression of BACE1 and/or APP, suggesting circKIAA1586 may influence Aβ pathology (Hébert et al. 2008; Long and Lahiri 2011; Pereira et al. 2016; Zhang et al. 2019). Other circRNAs also modulate amyloid pathways. CircCwc27, upregulated in the early stage of AD in mouse models and patient brains, has been observed to interact with Pur-α, altering transcriptional regulation of APP and promoting Aβ deposition (Song et al. 2022). Knocking out circCwc27 enhances the affinity of Pur-α for AD-associated genes promoters, such as APP, preventing Aβ deposition, reducing cognitive decline, and mitigating glial cell activation and pro-inflammatory cytokine production (Song et al. 2022). Among the most strongly AD-associated molecules there are also circHOMER1, a synaptic circRNA implicated in neuronal plasticity (Dube et al. 2019; Cervera-Carles et al. 2020; Lo et al. 2020). CircHOMER1 is downregulated in various brain regions of AD patients, including the olfactory and frontal cortex (Urdánoz-Casado et al. 2021), and its expression levels are negatively correlated with Aβ load and the pathological staging of AD (Cervera-Carles et al. 2020). Network analysis has shown a significant association between circHOMER1 and three key traits: AD patient status, Braak staging of NFTs, and clinical dementia rating **(**Beylerli et al. 2024). CircHOMER1 is highly expressed in the hippocampus and participates in synaptic transmission, plasticity, and neuronal excitation. Its downregulation contributes to the synaptic pathology seen in AD (You et al. 2015). It is also predicted to sponge miR-651 (Urdánoz-Casado et al. 2021), targeting AD-related genes PSEN1/PSEN2, while circCORO1C acts as a sponge for miR-105, thereby targeting the AD-related genes APP and α-synuclein (α-syn) gene (SNCA). These actions reduce the accumulation of Aβ and α-syn in neurons. Both circHOMER1 and circCORO1C expressions significantly correlated with AD neuropathology and clinical dementia ratings (Dube et al. 2019). Notably, circAβ-a, derived directly from the APP locus, can be translated into an Aβ-containing polypeptide that is further processed into Aβ peptides, suggesting an alternative source of amyloid production independent of canonical APP translation (Mo et al. 2020; Urdánoz-Casado et al. 2023). Another study establishes that circ_0004381 is significantly upregulated in AD models, where it contributes to neuronal damage and neuroinflammation. circ_0004381 sponges miR-647, thereby preventing miR-647 from suppressing its target, presenilin-1 (PSEN1). Knockdown of circ_0004381 was found to be neuroprotective, attenuating apoptosis and oxidative stress in hippocampal neurons and ultimately improving cognitive function in an AD mouse model (Li et al. 2022a). Finally, it is important to mention circMAPT, which is generated from the Tau gene locus (MAPT) and contains open reading frames that can encode Tau protein fragments, although their role in AD pathogenesis requires further study **(**Welden et al. 2018).

Beyond amyloid biology, circRNAs regulate neuroinflammation, a key driver of AD progression. Activated microglia releases pro-inflammatory cytokines that exacerbate neuronal damage (Katsumoto et al. 2018; Ahmad et al. 2019). CircPTK2 (circ_0008305) modulates microglial activation through miR-181c-5p and high mobility group protein B1 (HMGB1) signaling pathways (Table 1) (Li et al. 2021), and through miR-29b and the SOCS-1/JAK2/STAT3/IL-1β signaling pathway, which in turn controls microglial-mediated neuronal apoptosis (Wang et al. 2019). Dysregulated circRNAs such as circ_0000950 promote inflammatory cytokine expression and neuronal apoptosis in AD through the sponging of miR-103, which leads to increased expression of prostaglandin endoperoxide synthase 2 (PTGS2) (Yang et al. 2019b).

Oxidative stress and mitochondrial dysfunction represent additional pathological axes influenced by circRNAs in AD. For instance, the circPRKCI / miR-545/589 / E2F7 ceRNA network mediates neuronal damage induced by oxidative stress (Cheng et al. 2019). CircRNA-mediated networks involving Nrf2 signaling pathways have been also implicated in antioxidant responses (Yang et al. 2018a). Bioinformatics analyses suggest that several circRNAs, specifically mmu_circRNA_34132, mmu_circRNA_017077, and mmu-circRNA-015216, may be involved in Nrf2-mediated neuroprotection in a mouse model of AD, highlighting a potential role for circRNAs in modulating the cellular response to oxidative damage in AD (Kerr et al. 2017; Yang et al. 2018a). In another AD mouse model, mmu_circRNA_013636 was upregulated and mmu_circRNA_012180 was downregulated. Treatment with panax notoginseng saponins (PNS), which has anti-oxidative stress effects, reversed their expression changes, linking these circRNAs to oxidative stress pathways in AD (Huang et al. 2018a, b). CircRNAs further modulate autophagy and apoptosis, processes critical for clearing misfolded proteins and maintaining neuronal survival in AD. circRNA.2837 has been shown to induce neuronal autophagy by sponging the miR-34 family, and circHECTD1 can inhibit astrocyte activation by sponging miR-142 in an autophagy-dependent manner (Han et al. 2018; Zhou et al. 2018b). On the other hand, circSHOC2 regulates autophagy through the miR-7670-3p/SIRT1 axis to alleviate neuronal injury **(**Chen et al. 2020) reducing the levels of AD marker proteins tau and Aβ (Chen et al. 2020). Finally, circ_0003611 regulates apoptosis through miR-383-5p/KIF1B axis in AD (Table 1) (Li et al. 2022b).

Given their high synaptic enrichment, circRNAs are intimately linked to synaptic plasticity and transmission. Many are upregulated during synaptogenesis, and dysregulation correlates with synaptic vesicle cycling defects observed in AD (Mahmoudi and Cairns 2019). In addition to the already mentioned circHOMER1 (Dube et al. 2019), a recent study identified mmu_circ_017963 significantly dysregulated in an AD mouse model. Mmu_circ_017963 has been predicted to sponge mmu_miR_7033-3p and participate in biological processes related to autophagosome assembly (Huang et al. 2018a). Functional analysis revealed that mmu_circ_017963 is highly associated with the synaptic vesicle cycle process, indicating a potential role in modulating synaptic transmission and plasticity, which are severely compromised in AD (Huang et al. 2018a, 2020). Collectively, these findings illustrate that circRNAs are deeply integrated into the key pathological networks of AD, from Aβ and inflammation to synaptic failure. This intricate involvement is further compounded by the effects of aging. Aging itself profoundly reshapes the circRNAs landscape. Studies in aged mouse and primate brains reveal widespread circRNAs upregulation, likely reflecting both increased stability and age-related shifts in splicing factor fused in sarcoma (FUS) activity (Gruner et al. 2016; Errichelli et al. 2017; Kim et al. 2020). Modifications such as m6A may further regulate circRNA turnover in aging brain (Zhang et al. 2020a; Shafik et al. 2021). Age-associated circRNAs, including circGRIA1, have been linked to synaptic decline in the prefrontal cortex and hippocampus of male rhesus macaques (Xu et al. 2020). Knockdown of circGRIA1 leads to an improvement in synaptogenesis and synaptic plasticity, suggesting it contributes to synaptic decline with age (Xu et al. 2020). Astrocytes are also abundant brain cells whose senescence is implicated in aging and neurodegenerative disease. In astrocytes treated with amyloid-beta (Aβ), dysregulated circ_0006245 and circ_0004037 were predicted to sponge miRNAs that regulate extracellular matrix expression, cell proliferation, and apoptosis (Deng et al. 2022). Further compelling evidence comes from research using the senescence-accelerated mouse prone 8 (SAMP8) model, which is often used to study sporadic AD due to its age-related decline in learning and memory (Akiguchi et al. 2017). Interestingly, five circRNAs (mmu_circ_017963, mmu_circ_003540, mmu_circ_013699, mmu_circ_012180 and mmu_circ_006173) were found to be dysregulated when comparing the AD model mice to age-matched controls (Huang et al. 2018a). In conclusion, in SAMP8 model, subsets of aging-related circRNAs overlap with those dysregulated in AD, directly connecting molecular aging processes to neurodegenerative vulnerability. Collectively, these findings position circRNAs as central players in AD pathogenesis. They influence amyloid production and clearance, tau regulation, neuroinflammation, oxidative stress, autophagy, apoptosis, and synaptic plasticity. Their stability, brain enrichment, and disease-specific expression patterns make them promising candidates for diagnostic biomarkers and therapeutic targets. Importantly, their intersection with aging-related molecular changes suggests that circRNA dysregulation may help explain why advancing age remains the strongest risk factor for Alzheimer’s disease.

### circRNAs in Parkinson’s disease and amyotrophic lateral sclerosis pathogenesis

Parkinson’s disease (PD) is a chronic, progressive neurodegenerative disorder that most commonly emerges later in life, with overt motor symptoms frequently appearing in the sixth decade. Clinically, PD is characterized by bradykinesia, resting tremors, muscular rigidity, and postural instability, often accompanied by autonomic dysfunction, sleep disturbances, mood disorders, and cognitive decline. Pathologically, two cardinal features define the disease: (1) the abnormal accumulation of α-synuclein (encoded by the SNCA gene) and (2) the progressive degeneration of dopaminergic neurons within the substantia nigra pars compacta. Misfolded α-syn aggregates into intracellular inclusions known as Lewy bodies (LB), which are distributed throughout the central, peripheral, and enteric nervous systems. The selective vulnerability and loss of nigrostriatal dopaminergic neurons lead to profound dopamine depletion in the striatum, disrupting basal ganglia circuitry and ultimately driving the hallmark motor manifestations of PD (Smeyne and Smeyne 2013). At the molecular level, PD progression is tightly linked to dysregulated protein homeostasis, mitochondrial dysfunction, oxidative stress, impaired autophagy, neuroinflammation, and apoptotic signaling. In recent years, circRNAs have emerged as important regulators of these interconnected pathways. In PD, multiple circRNAs have been identified as either neuroprotective or neurotoxic, shaping core disease processes such as α-synuclein aggregation, neuroinflammation, apoptosis, and autophagy (Xiao et al. 2024). One of the most direct links between circRNAs and PD pathology involves circSNCA, which is derived from the 3′ untranslated region (UTR) of SNCA mRNA itself. CircSNCA regulates α-syn expression through its interaction with miR-7, a miRNA that normally suppresses SNCA translation. By sequestering miR-7, circSNCA reduces miR-7 availability, thereby permitting increased SNCA expression. Elevated circSNCA levels have been associated with increased expression of pro-apoptotic proteins, including CASP3, BAX, PTEN, and P53, alongside reduced levels of the autophagy marker LC3B-II, contributing directly to neurodegeneration (Table 2) (Sang et al. 2018; Titze-de-Almeida and Titze-de-Almeida 2023). In cellular PD models induced by the neurotoxin MPP+, circSNCA is upregulated in parallel with α-syn accumulation and apoptotic activation. Notably, treatment with the dopamine agonist pramipexole reduces circSNCA expression, increases free miR-7 levels, and decreases α-synuclein expression, linking a clinically used therapeutic agent to modulation of the ceRNA circSNCA/miR-7/SNCA network (Sang et al. 2018). These findings position circSNCA as both a mechanistic contributor to PD pathology and a potential therapeutic target. Similarly to circSNCA, CDR1as (ciRS-7), a well-known miR-7 sponge enriched in the brain, plays a significant role in maintaining miR-7 homeostasis. Knockout studies have demonstrated that disruption of CDR1as leads to miR-7 dysregulation and sensorimotor abnormalities in PD mice models, underscoring its importance in dopaminergic function (Piwecka et al. 2017). circHIPK3 also enhances neuroinflammation in PD models by sponging the neuroprotective microRNA miR-124 and regulating the miR-124-mediated STAT3/NLRP3 pathway in microglia (Zhang et al. 2022a; b). CircPank1 also promotes dopaminergic neurodegeneration when upregulated, enhancing α-synuclein expression through sequestration of miR-7a-5p () (Liu et al. 2022a; He et al. 2022). On the other hand, in a study conducted in a *C. elegans* model of PD, circ_zip-2 has been identified as a protective molecule that is significantly downregulated in the disease state. Knockdown of this circRNA reduced the aggregation of α-synuclein, a hallmark of PD (Kumar et al. 2018). It functions by binding to miR-60, thereby mitigating the pathological process. This protective role has been shown to directly affect two core features of PD pathology: it reduces the aggregation of SNCA and lowers the content of damaging reactive oxygen species (ROS), highlighting its role in counteracting cellular stress (Kumar et al. 2018; Wu et al. 2023; Xiao et al. 2024).

**Table 2:** PD- and ALS-related circRNAs

Gene	Trend	circRNA Function	Target gene and Functional Pathway	Source	Functional activity	References
ciRS-7 (or CDR1as)	Down	miRNA sponges	CDR1as↓ → miR-7↑ → Fos↓ → sensorimotor alterations	CDR1as-KO mouse model of PD	Knockout leads to miR-7 dysregulation.	(Piwecka et al. 2017)
circ_0004381	Up	miRNA sponges	circ_0004381↑ → miR-185-5p↓ → RAC1↑ → inflammatory response↑, oxidative stress↑ → cell viability↓, apoptosis↑	Cellular PD model of MPP+ -induced SH-SY5Y neuroblastoma	Induces neuron injury	(Zhang et al. 2022b)
circSNCA	Up	miRNA sponges	circSNCA↑ → miR-7↓ → SNCA↑ → pro-apoptotic proteins: CASP3, BAX, PTEN and p53 ↑ → anti-apoptotic protein BCL2↓ → autophagy-associated protein LC3B-II ↓ → neurodegeneration↑	Cellular PD model of MPP+ -induced SH-SY5Y neuroblastoma	Associated with neurodegeneration. Promising and specific therapeutic target for PD treatment.	(Sang et al. 2018).
circ_0070441	Up	miRNA sponges	circ_0070441↑ → miR-626↓ → IRS2↑ → cellular Aβ level↑	MPP+ -induced cellular PD model	Induces neurotoxicity, facilitator of neuronal damage in PD	(Xie et al. 2021; Cao et al. 2022).
circHIPK3	Up	miRNA sponges	circHIPK3↑ → miR-124↓ → STAT3/NLRP3↑ → IL-6, IL-1β and TNF-α↑	Sera and CSF of PD patients and *in vitro* microglia model	Enhances neuroinflammation	(Zhang et al. 2022a; d).
circSLC8A1	Up	miRNA sponges and Scaffolding RNA-Binding Protein	Binds to the protein AGO2, essential protein of RISC; circSLC8A1↑ → miR-128↓ → SIRT1, BMI1 and Axin1↑ → dopaminergic neurons apoptosis↑	Postmortem brain SN from PD patients and cellular PD model	Induced by oxidative stress and neuroinflammation. Involved in dopaminergic neurons apoptosis	(Hanan et al. 2020).
circDLGAP4 (circ_0060180)	Down	miRNA sponges	circDLGAP4↓ → miR-134-5p↑ → CREB↓ → neuronal damage ↑, viability↓, apoptosis↑, mitochondrial damage↑, autophagy↓ →neuroprotection↓	Human brain of PD patients, mouse PD model and MPP+ -induced cellular PD model	Overexpression has a neuroprotective effect decreasing apoptosis	(Bai et al. 2018; Feng et al. 2020).
circSAMD4A	Up	miRNA sponges	circSAMD4A↑ → miR-29c-3p↓ → AMPK/mTOR↑ → apoptosis and autophagy ↑	MPP+ neurotoxin-induced PD cell models	Pro-apoptotic and pro-autophagic effect in dopaminergic neurons	(Wang et al. 2021; Song et al. 2021).
circ_zip-2	Down	miRNA sponges	cirzip2↓ → miR-60-3p↑ → α-syn↓ → α-synuclein aggregation↓ → ROS↓	*C. elegans* model of PD	Knockdown reduces the aggregation of α-synuclein, a hallmark of PD	(Kumar et al. 2018).
circTLK1	Up	miRNA sponges	circTLK1↑ → miR-26a-5p↓ → DAPK1↑ → apoptosis↑, neuronal injury↑	PD mouse models	Knockdown inhibits apoptosis. Promising therapeutic target	(Chen et al. 2022).
circPank1	Up	miRNA sponges	circPank1↑ → miR-7a-5p↓ → α-syn↑ → dopaminergic neuron neurodegeneration↑	SN of PD model mice and MN9D cell model of dopaminergic neurons	Promotes dopaminergic neurodegeneration	(Liu et al. 2022a)
circ_0000497 (circSLAIN1)/ circ_0000826 (circANKRD12)/ circ_0003848 (circPSEN1)	Up	Unknown	Unknown	PBMC of PD patients	High diagnostic sensitivity and specificity	(Ravanidis et al. 2021)
circ-Hdgfrp3	Down	Recruiting RNA-Binding Protein	circ-Hdgfrp3↓ → cytoplasmic FUS↓	Motor neurons cell line	Maintains neuronal integrity. In some ALS cases, mtFUS affects its localization	(D’Ambra et al. 2021).
circ_0023919 (circPICALM)/ circ_0088036 (circSUSD1)/ circ_0063411 (circTNRC6B)	Up	Unknown	Unknown	Peripheral blood of ALS patients	90% of Sensitivity and specificity. Potential blood-based diagnostic biomarkers	(Dolinar et al. 2019)

*Abbreviation*. *Up* up-regulated, *Down* down-regulated, *miRNA* microRNA, *KO* knockout, *PD* Parkinson’s disease, *RAC1* Ras-related C3 botulinum toxin substrate 1, *MPP* 1-methyl-4-phenylpyridinium neurotoxin, *SNCA* α-synuclein gene, *CASP3* caspase-3, *BAX* BCL2-associated X protein, *PTEN* phosphatase and tensin homolog, *BCL-2 *B-cell lymphoma 2, *LC3B-II* lipidated, membrane-bound form of microtubule-associated protein 1A/1B-light chain 3B (LC3B), *IRS2* insulin receptor substrate 2, *Aβ* amyloid beta peptide, *STAT3* signal transducer and activator of transcription 3, *NLRP3 *NOD-like receptor protein 3, *IL* interleukin, *TNF* tumor tecrosis factor, *CSF* cerebrospinal fluid, *RISC* RNA-induced silencing complex, *AGO2* argonaute-2, *BMI1* B lymphoma Mo-MLV insertion region 1 homolog, *Axin1* axis inhibition protein 1, *SN* substantia nigra, *CREB *cAMP response element-binding protein, *AMPK* AMP-activated protein kinase, *mTOR* mammalian target of rapamycin, *ROS *reactive oxygen species, *α-syn *α-synuclein, *DAPK1 *death-associated protein kinase 1, *PBMC* peripheral blood mononuclear cells, *FUS* fused in sarcoma protein, *ALS* amyotrophic lateral sclerosis, *mtFUS* mutant FUS protein

Beyond protein aggregation, circRNAs also regulate neuroinflammatory cascades. CircHIPK3 is upregulated in PD models and enhances microglial activation by sponging miR-124, a microRNA with well-established anti-inflammatory and neuroprotective functions. Through the miR-124/STAT3/NLRP3 pathway, circHIPK3 promotes inflammatory cytokine release and exacerbates neuronal injury (Table 2) (Zhang et al. 2022a; b). Given the growing recognition that chronic microglial activation contributes to disease progression, circRNA-driven inflammatory amplification may represent a key pathogenic mechanism. Oxidative stress, another major driver of dopaminergic vulnerability, is also modulated by circRNAs. CircSLC8A1 has been shown to be significantly elevated in the substantia nigra of PD patients and is inducible by oxidative stress *in vitro* (Hanan et al. 2020). Mechanistically, circSLC8A1 interacts with AGO2 and contains multiple binding sites for miR-128. By modulating miR-128 activity, circSLC8A1 influences the expression of genes involved in neuronal survival, aging, and stress responses, including SIRT1, BMI1, and Axin1 (Chatoo et al. 2009; Min et al. 2013; Zhou et al. 2018a). Elevated circSLC8A1 levels correlate with enhanced oxidative damage, whereas neuroprotective interventions reduce its expression, suggesting that the circSLC8A1/miR-128 axis participates in redox imbalance and dopaminergic degeneration (Hanan et al. 2020).

Autophagy and apoptosis, two tightly linked processes governing neuronal fate, are also subject to circRNA regulation in PD. CircSAMD4A is upregulated in MPP+-induced PD models and promotes both apoptosis and autophagy of dopaminergic neurons via sponging miR-29c-3p and modulating the AMPK/mTOR signaling pathway (Table 2) (Song et al. 2021). Although autophagy is generally protective, dysregulated or excessive autophagy can contribute directly to the neurodegenerative process observed in PD **(**Wang et al. 2021). Similarly, circTLK1 is significantly increased in PD mouse models and promotes neuronal injury through the circTLK1/miR-26a-5p/death-associated protein kinase 1 (DAPK1) axis. Experimental knockdown of circTLK1 reduces apoptosis and improves neuronal viability, highlighting its potential as a therapeutic target (Chen et al. 2022). Conversely, CircDLGAP4 is downregulated in both mouse and cell models of PD, and it has been demonstrated to have a clear neuroprotective effect decreasing apoptosis and improving cell survival (Bai et al. 2018; Feng et al. 2020; Valizadeh et al. 2024). It functions by sponging miR-134-5p and activating the CREB signaling pathway, a critical regulator of neuronal plasticity and survival (Bai et al. 2018). Overexpression of circDLGAP4 mitigates MPP+-induced damage in cellular PD models, suggesting that loss of this circRNA removes a neuroprotective brake (Feng et al. 2020). Originating from the SNCA gene, circ_0070441 is upregulated in PD cell models and exerts a neurotoxic effect (Sang et al. 2018). It functions by adsorbing miR-626, which leads to an upregulation of insulin receptor substrate 2 (IRS2), which affects the cellular Aβ level. This mechanism has been shown to aggravate the neurotoxicity triggered by MPP+, marking circ_0070441 as a facilitator of neuronal damage in PD **(**Xie et al. 2021; Cao et al. 2022). Collectively, these findings reveal a complex and often antagonistic circRNA landscape in PD. Some circRNAs are upregulated and actively promote neurodegeneration by enhancing α-syn accumulation, inflammation, oxidative stress, and apoptosis. Others are downregulated, and their loss removes protective regulatory constraints on stress-response and survival pathways. Across these examples, the predominant mechanism involves miRNA sequestration, linking circRNAs to major pathogenic axes such as SNCA regulation, AMPK/mTOR-mediated autophagy, CREB-dependent neuroprotection, and inflammatory signaling. Briefly, similar principles extend to other neurodegenerative disorders such as amyotrophic lateral sclerosis (ALS), which is characterized by motor neuron degeneration and aggregation of TDP-43. In ALS, circRNAs interact with RNA-binding proteins such as FUS (Kim et al. 2020); mutant FUS alters circ-Hdgfrp3 localization and disrupts neuronal homeostasis. Circ-Hdgfrp3 contributes to maintaining neuronal integrity, and its dysregulation is linked to abnormal cytoplasmic FUS accumulation (D’Ambra et al. 2021). Moreover, several dysregulated circRNAs have been identified in ASL patient blood samples: pecifically, four circRNAs (circ_0000567, circ_0023919, circ_0088036, and circ_0063411) have demonstrated sensitivity and specificity higher than 90% as potential blood-based diagnostic biomarkers, outperforming existing markers (Dolinar et al. 2019).

## Biological functions of circRNAs in viral infections

CircRNAs regulate host inflammatory responses and viral life cycles through multiple, mechanistically distinct pathways, positioning them as central modulators of infection biology. Far from being inert byproducts of splicing, circRNAs are deeply integrated into innate immune signaling networks and can both activate and be shaped by antiviral defense pathways. One fundamental layer of interaction involves innate immune sensing. Exogenous or “foreign” circRNAs can be detected by cytosolic pattern-recognition receptors such as Retinoic Acid–Inducible Gene I (RIG-I) and Protein Kinase R (PKR). Compared with linear RNAs of identical sequence, certain unmodified circRNAs elicit a stronger innate immune response, leading to robust induction of type I interferons and interferon-stimulated genes (Chen et al. 2017). To evade such detection, endogenous circRNAs often undergo m6A modification, which reduces recognition by RIG-I and dampens immune activation (Chen et al. 2019). During viral infection, activation of RNase L, an interferon-induced endoribonuclease, leads to widespread degradation of cellular circRNAs (Zheng 2019**)**. Many circRNAs contain double-stranded stem–loop structures that normally bind and sequester inactive PKR. Their degradation releases PKR, enabling its activation and amplification of antiviral signaling. Similarly, reduced circRNA abundance can release nuclear factors such as NF90/NF110, which translocate to the cytoplasm and bind viral RNAs, suppressing viral replication. Thus, circRNA turnover itself becomes an active component of the host antiviral response (Li et al. 2017). Both host cells and viruses exploit circRNAs during infection. Host-derived circRNAs frequently function as miRNA sponges and, in a context of viral infection and virus-associated malignancies, this mechanism can profoundly influence inflammation, immune evasion, and tumor progression (Yin et al. 2024; Gou et al. 2025). In Hepatitis B virus (HBV)-associated hepatocellular carcinoma (HCC), several host circRNAs drive tumor development by sponging tumor-suppressive miRNAs. Axes such as circRNA_101764/miR-181, regulate proliferation, invasion, and metastasis (Table 3) (Liao et al. 2021). Additionally, circ_0004812 is upregulated in HBV-infected patients, where it binds miR-1287-5p to increase follistatin-like protein 1 (FSTL1) expression, attenuating interferon-mediated antiviral immunity (Zhang and Wang 2020). In Epstein-Barr virus (EBV)-associated nasopharyngeal carcinoma, host circCRIM1 is upregulated and sponges miR-422a, promoting metastasis and chemoresistance (Hong et al. 2020). CircRNAs also shape inflammatory responses in acute viral infections. During Japanese encephalitis virus (JEV) infection, circ_0000220 sponges miR-326-3p, increasing inflammatory cytokine production (Li et al. 2020a). Enterovirus 71 (EV-A71) infection induces circ_0045431, which activates the NLRP3 inflammasome through miR-584 sequestration, promoting pyroptosis and influencing viral replication (Hu et al. 2023). Beyond miRNA sponging, circRNAs directly interact with proteins. They can function as scaffolds facilitating protein-protein interactions or as decoys that sequester viral or host proteins. During Influenza A virus (IAV) infection, circVAMP3 binds viral nucleoprotein (NP) and nonstructural protein 1 (NS1), disrupting replication complexes and restoring innate immune signaling (Min et al. 2023). In contrast, circMerTK suppresses interferon-β activation, weakening antiviral defenses and facilitating viral propagation (Qiu et al. 2023). In Kaposi’s sarcoma-associated herpesvirus (KSHV) latency, circ_0001400 interacts with the splicing factor PNISR to suppress viral gene expression and inhibit apoptosis, thereby stabilizing the latent state (Tagawa et al. 2023). In human immunodeficiency virus (HIV-1) infection, the viral protein Vpr hijacks the host circRNA ciTRAN to displace SRSF1 from transcriptional complexes, enhancing viral gene expression (Bhardwaj et al. 2023). A notable example of circRNA-mediated antiviral defense involves circ_29164 in Pseudorabies virus (PRV) infection. PRV, isolated from human patients with ophthalmitis or encephalitis (He et al. 2019), has been shown to be inhibited by circ_29164 overexpression. Mechanistically, circ_29164 sponges ssc-miR-24-3p, upregulating Kelch-like ECH-associated protein 1 (KEAP1), which promotes apoptosis as an antiviral defense. This circ_29164/miR-24-3p/KEAP1 axis represents a defined molecular cascade linking circRNA regulation to programmed cell death and viral restriction (Li et al. 2024a). Indirectly, dysregulated apoptosis in patients with ophthalmitis or encephalitis caused by PRV may represent a risk factor for the development of neurodegeneration as a consequence of viral infection sequelae.

**Table 3:** circRNAs involved in neurotropic viral infection

Virus	CircRNA	Disease	Potential Role/Mechanism	
SARS-CoV-2	circ_3205 (viral encoded)	COVID-19	Diagnostic marker; sponges hsa-miR-298, upregulating PRKCE and KCNMB4 to promote infection.	(Barbagallo et al. 2022)
SARS-CoV-2	circ_00022_SMC1A/ circ_00007_MAN1A2	COVID-19-associated neurological manifestations	Significantly downregulated in CSF of patients affected by COVID-19-associated neurological manifestations	(Reinhold et al. 2023)
IAV	circVAMP3	Influenza	Directly disrupting the formation of RNP complexes and blocking viral replication by decoying viral proteins NP and NS1.	(Min et al. 2023)
IAV	circMerTK	Influenza	Suppresses the activation of IFN-β, weakening the host’s antiviral response and aiding viral replication.	(Qiu et al. 2023)
ZIKA	circ_0007321	Congenital Zika Syndrome	Regulates ZIKV replication via the miR-492/NFKBID/NF-κB pathway.	(Kang et al. 2023)
HIV-1	ciTRAN	AIDS	Hijacked by HIV-1 to promote effective viral transcription.	(Bhardwaj et al. 2023)
DENV	circ_0006459, circ_0015962	Dengue Fever	Levels change significantly before and after treatment.	(He et al. 2019 a)
JEV	circ_0000220	JEV-associated encephalitis	Sponges miR-326-3p, leading to increased expression of inflammatory cytokines	(Li et al. 2020a).
EV-A71	circ_0045431	EV-A71 associated Neuropathological damage	Induced by viral infection, sponges hsa_miR_584 to activate the NLRP3 inflammasome.	(Hu et al. 2023)
PRV	circ_29164	Ophthalmitis and encephalitis	Its antiviral effect is mediated by the induction of apoptosis.	(He et al. 2019 b; Li et al. 2024a).

*Abbreviations. SARS-CoV-2* severe acute respiratory syndrome coronavirus 2, *COVID-19 *coronavirus disease 2019, *PRKCE* protein kinase C epsilon, *KCNMB4* potassium calcium-activated channel subfamily M regulatory beta subunit 4, *IAV *influenza A Virus, *RNP* ribonucleoprotein, *NP *nucleoprotein, *NS* nonstructural protein 1, *MerTK* MER proto-oncogene, Tyrosine Kinase, *IFN-β* type I interferon beta, *ZIKA *Zika virus, *NFKBID* nuclear factor kappa-B inhibitor delta, *NF-κB *nuclear factor kappa B, *HIV-1* human immunodeficiency virus, *AIDS* acquired immunodeficiency syndrome, *DENV* Dengue virus, *JEV* Japanese encephalitis virus, *EV-A71* enterovirus A71, *NLRP3 *NOD-like receptor protein 3*, PRV *pseudorabies virus

Viruses themselves encode circRNAs (vcircRNAs) that promote immune evasion, persistence, and oncogenesis. Some vcircRNAs contain IRES enabling translation. In human papillomavirus (HPV)-16 positive cervical cancer cells, virus-derived circE7 produces the E7 oncoprotein, directly contributing to carcinogenesis (Zhao et al. 2019; Wang et al. 2022). Severe acute respiratory syndrome coronavirus 2 (SARS-CoV-2)-derived circ_3205, originating from the nucleocapsid gene, sponges hsa-miR-298, increasing PRKCE and KCNMB4 expression to favor viral progression (Table 3) (Barbagallo et al. 2022). Together, these findings reveal circRNAs as dynamic regulators at the interface of viral replication and host immunity. Whether functioning as immune activators, molecular decoys, miRNA sponges, protein scaffolds, or even translated viral products, circRNAs constitute a versatile regulatory layer that shapes infection outcomes, inflammation, latency, and virus-associated oncogenesis.

## circRNAs as potential diagnostic and prognostic biomarkers in NDDs and viral infections

CircRNAs, a class of endogenous non-coding RNAs known for their high stability and tissue-specific expression, are emerging as highly promising biomarker candidates. In AD, a pivotal observation demonstrated that circRNA expression levels correlate strongly with both clinical severity and neuropathological burden. Importantly, certain circRNA alterations can be detected before the onset of overt symptoms, suggesting that they may serve as non-invasive peripheral biomarkers capable of identifying individuals in pre-symptomatic stages and opening a crucial window for early intervention (Dube et al. 2019). Profiling studies in cerebrospinal fluid (CSF) have reinforced this potential. Microarray analyses comparing AD patients and healthy controls identified 163 dysregulated circRNAs. Subsequent RT-qPCR validation confirmed significant expression changes in multiple candidates. Among them, circ-AXL, circ-GPHN, circ-ITPR3, circ-LPAR1, and circPCCA emerged as independent predictors of AD risk and potential indicators of disease progression. Their levels correlated significantly with Mini-Mental State Examination (MMSE) scores and with core CSF biomarkers, including amyloid-β1–42 and tau proteins (Table 1) (Li et al. 2020b). Notably, circPCCA appears to exert functional relevance by sponging miR-138-5p and attenuating tau phosphorylation, thereby potentially mitigating disease severity (Wang et al. 2015). These findings underscore the clinical utility of circRNAs in CSF for risk stratification, disease monitoring, and personalized patient management. Additional studies have identified circ_0001535 as a promising diagnostic marker, potentially acting through an E2F1/DHFR regulatory axis (Ma et al. 2023). CircCwc27 and other circRNAs are upregulated even before amyloid plaque deposition, supporting their value as preclinical indicators (Song et al. 2022). Meanwhile, circHOMER1 levels correlate with disease severity, suggesting a role in staging and progression assessment (Dube et al. 2019; Cervera-Carles et al. 2020; Lo et al. 2020).

Beyond CSF, circRNAs are highly stable in blood, enhancing their appeal as minimally invasive biomarkers. In peripheral blood mononuclear cells (PBMC) from AD patients, 4,060 circRNAs were found to be differentially expressed, with nearly equal proportions up- and downregulated (Li et al. 2020c). Functional enrichment analyses linked these circRNAs to biological processes central to AD pathogenesis, including inflammation, immune regulation, metabolism, and neuronal development. Key pathways implicated included MAPK, mTOR, AMPK, and WNT signaling (Li et al. 2020c). CeRNA network modeling identified three upregulated circRNAs (circ_101618, circ_405619, and circ_000843) as potential regulators of 15 miRNAs and 223 target mRNAs. A particularly complex regulatory hub involved miR-455-3p, a miRNA previously proposed as an AD biomarker, with 13 dysregulated circRNAs predicted to contain its response elements (Li et al. 2020c).

Several altered circRNAs originate from genes linked to AD biology. For instance, circ_405619 and circ_000843 derive from PRKCA and PIK3C3, both involved in amyloid precursor protein (APP) processing (Table 1). Others map to VRK2 (circ_402265), DNMT3A (circ_405836) and APP (circ_061346 and circ_061343) reinforcing their pathophysiological relevance. Interestingly, circ_402265 and circ_405836 were among the top 10 most significantly upregulated and downregulated circRNAs, respectively, in PBMC from AD patients (Li et al. 2020c; Ghafouri-Fard et al. 2022). Additionally, hsa_circ_0003391 is significantly downregulated in AD blood samples compared with healthy controls and other dementia types. Its levels correlate with cognitive decline and hippocampal atrophy, and it may function through sponging miR-574-5p, further highlighting its diagnostic and mechanistic relevance (Liu et al. 2020).

In PD, circRNAs similarly demonstrate diagnostic promise. Initial studies identified four PBMC-derived circRNAs (SLAIN1_circ_0000497, ANKRD12_circ_0000826, and PSEN1_circ_0003848) with high sensitivity and specificity (Table 2) (Ravanidis et al. 2021). More recently, large-scale analyses revealed 192 differentially expressed circRNAs in PD blood (Beric et al. 2024). This included circRHBDD1, which was previously reported to be downregulated in PD blood (Whittle et al. 2024), and three circRNAs (circCSE1L, circRNF13, circSHOC2) previously found to be dysregulated in brain tissue (Dong et al. 2023). Moreover, a validated subset of nine high-confidence circRNAs (circAFF2, circCCDC91, circETFA, circFAM13B, circITGAX, circNCF1, circPADI4, circSPI1, circSUZ12) was consistently dysregulated across cohorts (Beric et al. 2024). Notably, altered circRNA levels were detected in at-risk individuals prior to symptom onset, including non-manifesting mutation carriers. Several circRNAs correlated with motor severity, and circAFF2 was associated with cognitive status measured by the Montreal Cognitive Assessment (MoCA). Functional analyses linked these circRNAs to dopaminergic synapse regulation, Hippo signaling, and ubiquitin-mediated proteolysis, aligning with established PD pathobiology (Beric et al. 2024).

Collectively, these findings provide compelling evidence that circRNAs represent a robust and minimally invasive class of biomarkers for both AD and PD. Their stability, detectability in accessible biofluids, correlation with disease severity, and reflection of underlying molecular pathways underscore their potential for early diagnosis, progression monitoring, and improved understanding of neurodegenerative disease mechanisms.

CircRNAs are also highly stable and can be readily detected in CSF, plasma, and exosomes during viral infections, offering minimally invasive tools for disease detection and monitoring therapeutic responses. Recently, two circRNAs (circ_00022_SMC1A and circ_00007_MAN1A2) were found to be significantly downregulated in CSF of patients with neurological manifestations associated with COVID-19, compared with both non-neuroinflammatory controls and patients with NDDs (Table 3) (Reinhold et al. 2023). These observations strongly suggest that the circRNA profile in the CSF of patients with neurological manifestations associated with neurotropic viral infections may represent a valid approach for investigating the close relationship between neuroinflammation and neurodegeneration. On the other hands, exosomal circRNAs are increasingly being investigated as biomarkers in virus-related malignancies, including those associated with HBV, Hepatitis C virus (HCV), EBV, and HPV (Zhang et al. 2023; Saadh et al. 2023). 

## circRNAs as novel therapeutic targets in NDDs and viral infections

The remarkable stability, cell-type specificity, and functional relevance of circular RNAs (circRNAs) make them compelling candidates for therapeutic intervention in neurodegenerative disorders and viral infections. Because specific circRNAs are dysregulated during the preclinical stages of AD, PD, and several viral infections, strategies aimed at restoring or inhibiting these molecules could potentially halt or slow disease progression at a time when neuronal dysfunction is still limited. Early molecular intervention may therefore represent one of the most promising applications of circRNA-based therapeutics. Despite this potential, several challenges must be addressed, particularly with respect to safety, delivery efficiency, and tissue specificity. Targeting the brain remains a central obstacle. One promising strategy involves extracellular vesicles (EVs) engineered to express rabies virus glycoprotein (RVG), which enables selective targeting of neuronal cells. RVG-modified EVs have successfully delivered therapeutic circRNAs, such as circSCMH1 in stroke and circDYM in depression animal models, demonstrating effective brain uptake and functional benefit *in vivo* (Yang et al. 2020; Yu et al. 2022). Loss-of-function approaches require highly specific targeting to avoid unintended suppression of linear host transcripts. Small interfering RNAs (siRNAs) or short-hairpin RNAs (shRNAs) must be designed against the unique back-splice junction (BSJ) of circRNAs, ensuring selectivity. Antisense oligonucleotides (AONs) represent another strategy: these single-stranded molecules bind complementary circRNA sequences, promoting degradation or blocking protein-binding sites. CRISPR/Cas-based technologies further expand therapeutic options: precise deletion of circRNA-specific BSJ sequences can eliminate circRNA expression without disrupting the linear transcript, thereby minimizing off-target effects (Wu et al. 2023). Conversely, gain-of-function strategies aim to restore or enhance neuroprotective circRNAs. Viral and non-viral vectors, including adenoviral and lentiviral systems, can be engineered with exon-intron cassettes that promote back-splicing and circRNA overexpression (Liang and Wilusz 2014; Chen et al. 2017; Mecozzi et al. 2022). For example, synthetic siRNA-mediated silencing of circSLC8A1, a contributor to PD pathology, modulates miR-128 activity and ameliorates pathogenic pathways in cellular models (Hanan et al. 2020). In AD, restoring protective circRNAs such as ciRS-7 or circHDAC9, or inhibiting pathogenic ones, represents also a rational therapeutic direction (Zhang et al. 2020c). However, beyond their established role as microRNA sponges, other circRNA functions, including protein scaffolding, peptide translation, and transcriptional regulation, remain insufficiently explored and warrant deeper investigation.

In the antiviral field, circRNAs are also emerging as versatile therapeutic platforms. Targeted inhibition of pathogenic circRNAs, such as circATP5H in HBV-HCC, suppresses viral replication (Jiang et al. 2020). Artificial circRNAs engineered to sponge essential host factors, such as miR-122 in HCV infection, effectively reduce viral replication *in vitro* (Jost et al. 2018). Moreover, engineered circRNAs encoding viral antigens are being developed as next-generation vaccines. Compared with linear mRNA vaccines, circRNA vaccines offer enhanced stability, prolonged antigen expression, and potentially reduced immunogenicity (Wesselhoeft et al. 2018, 2019; Liu et al. 2022b). A circRNA vaccine developed in 2022 demonstrated strong protection against SARS-CoV-2 and its variants in both mice and rhesus monkeys (Qu et al. 2022). Recently, Zhou and colleagues developed four monovalent circRNAs that encode monkeypox Virus (MPXV) antigens: cirA29L, cirA35R, cirB6R, and cirM1R. These circRNAs can elicit comprehensive and effective protection against the MPVX (Zhou et al. 2024). Finally, circRNA vaccines encoding different subtype of neuraminidase (NA) antigens from H1N1, H3N2, and influenza B viruses can induce broad-spectrum NA immunity against heterologous influenza strains, which is highly important for the development of broad-spectrum vaccines (Yue et al. 2024). Beyond antigen expression, exogenous circRNAs can incorporate immunomodulatory molecules, functioning similarly to vaccine adjuvants. For instance, circRNAs co-expressing CXCL13 and influenza hemagglutinin enhanced cross-reactive antibody production when delivered via lipid nanoparticles (Wan et al., 2024).

Although significant hurdles remain, including delivery optimization, off-target risk, and manufacturing costs, circRNA-based biotechnology represents a transformative and rapidly expanding frontier for treating neurodegenerative diseases and viral infections.

## Conclusion and future directions

Despite the rapid expansion of circRNA research in both neurodegeneration and virology, these fields have largely progressed in parallel rather than in integration. Several studies have characterized circRNA dysregulation in Alzheimer’s disease, Parkinson’s disease,, and other neurodegenerative disorders. A separate growing body of literature has demonstrated that circRNAs modulate viral replication, antiviral immunity, latency, and virus-associated oncogenesis. However, there are currently no comprehensive studies or public data sets that directly examine circRNA alterations in the brain following neurotropic viral infection within the context of neurodegeneration. Thus, while circRNAs independently intersect with both viral infection and neurodegeneration, the integrated virus-circRNA-neurodegeneration axis remains largely unexplored. This knowledge gap represents both a limitation and an opportunity. High-throughput sequencing technologies have generated extensive catalogs of circRNAs associated with brain aging, neurodegenerative pathology, and viral infection. Yet the specific functions of most identified circRNAs remain unknown: It is essential to determine how individual circRNAs influence cellular senescence, protein aggregation, neuroinflammation, synaptic integrity, and neuronal survival in NDDs and neurotropic viral Infections. Moving forward, research must prioritize functional validation to translate basic discoveries into therapeutic applications.

CircRNAs also hold significant promises as clinical biomarkers. Their high stability and detectability in bodily fluids, including blood and cerebrospinal fluid (CSF), make them attractive candidates for early diagnosis, prognosis, and therapeutics in NDDs. If robust and reproducible circRNA signatures can be identified, they may provide minimally invasive tools to detect disease onset, progression, or reveal prior exposure to neurotropic viral infection. Beyond diagnostics, circRNAs possess therapeutic potential. Their stability, relatively low immunogenicity, and modular structure make them suitable platforms for engineered RNA therapeutics, including protein expression systems or immune-modulating constructs. Conjugated bioactive molecules packaged within exosomes and coupled to stable circRNA scaffolds could, in principle, enhance delivery across the blood–brain barrier and target specific neural populations. The study of virus-encoded circRNAs represents another promising frontier. Many viruses generate their own circRNAs, yet most of their functions remain poorly characterized. Integrating circRNA biology with classical virology may reveal how viruses have co-opted or evolved circular RNA-based strategies to evade immunity, establish latency, or manipulate host transcriptional programs. Identifying such mechanisms could expose novel therapeutic vulnerabilities across viral infection.

Nevertheless, substantial challenges remain. Current evidence derives from preclinical models, often limited to *in vitro* systems or small animal studies with large-scale validation in human cohorts still lacking. Many mechanistic insights have been obtained from blood-based analyses, with insufficient data from lesion-specific brain regions or other relevant tissues. Furthermore, circRNA-mediated regulatory networks involving miRNAs, mRNAs, RBPs, and innate immune sensors are highly complex and require rigorous experimental confirmation (Li et al. 2024b). Future research directions are clear. Three-dimensional *in vitro* models of neurotropic viral infection, such as brain organoids, should be used to profile host and viral circRNAs under controlled conditions. Complementary *in vivo* models of acute, chronic, or latent infection are needed to assess circRNA expression across specific brain regions and correlate these changes with neurodegenerative markers, including protein aggregation, neuroinflammation, and neuronal loss. Post-mortem analyses of rare human cases involving both neurotropic infection and neurodegeneration would provide critical translational relevance. Functional studies also must follow expression profiling. For circRNAs significantly altered after viral infection, knockdown and overexpression strategies, using siRNA, shRNA, CRISPR-based approaches, or circRNA expression vectors, can determine whether these molecules influence protein misfolding, apoptosis, inflammatory signaling, or synaptic dysfunction. Mechanistic dissection should clarify whether their actions depend on microRNA sponging, protein binding, transcriptional modulation, splicing regulation, or interaction with innate immune sensors.

In summary, circRNAs represent a paradigm shift in molecular neuroscience and infection biology. Their abundance, stability, cell-type specificity, and functional diversity position them as powerful regulators at the intersection of aging, neurodegeneration, and viral pathogenesis. Although significant hurdles remain, continued multidisciplinary investigation holds the promise of unlocking novel diagnostic tools and RNA-based therapeutic strategies capable of transforming the management of neurodegenerative diseases and neurotropic viral infections.

## Data Availability

No datasets were generated or analysed during the current study.

## References

[CR1] Abdelmohsen K, Panda AC, Munk R et al (2017) Identification of HuR target circular RNAs uncovers suppression of PABPN1 translation by *CircPABPN1*. RNA Biol 14:361–369. 10.1080/15476286.2017.1279788 28080204 10.1080/15476286.2017.1279788 PMC5367248

[CR2] Ahmad MH, Fatima M, Mondal AC (2019) Influence of microglia and astrocyte activation in the neuroinflammatory pathogenesis of Alzheimer’s disease: Rational insights for the therapeutic approaches. J Clin Neurosci 59:6–11. 10.1016/j.jocn.2018.10.034 30385170 10.1016/j.jocn.2018.10.034

[CR3] Akiguchi I, Pallàs M, Budka H et al (2017) SAMP8 mice as a neuropathological model of accelerated brain aging and dementia: Toshio Takeda’s legacy and future directions. Neuropathology 37:293–305. 10.1111/neup.12373 28261874 10.1111/neup.12373

[CR4] Aktaş T, Avşar Ilık İ, Maticzka D et al (2017) DHX9 suppresses RNA processing defects originating from the Alu invasion of the human genome. Nature 544:115–119. 10.1038/nature21715 28355180 10.1038/nature21715

[CR5] Alzheimer’s Association A (2025) 2025 Alzheimer’s disease facts and figures. Alzheimers Dement 21:e70235. 10.1002/alz.70235

[CR6] Amelimojarad M, Amelimojarad M (2025) Regulatory mechanism of circular RNAs in brain and neurodegenerative diseases. Front Mol Neurosci 18:1507575. 10.3389/fnmol.2025.1507575 40453757 10.3389/fnmol.2025.1507575 PMC12123696

[CR7] Ashwal-Fluss R, Meyer M, Pamudurti NR et al (2014) circRNA Biogenesis Competes with Pre-mRNA Splicing. Mol Cell 56:55–66. 10.1016/j.molcel.2014.08.019 25242144 10.1016/j.molcel.2014.08.019

[CR8] Aubry S, Shin W, Crary JF et al (2015) Assembly and Interrogation of Alzheimer’s Disease Genetic Networks Reveal Novel Regulators of Progression. PLoS ONE 10:e0120352. 10.1371/journal.pone.0120352 25781952 10.1371/journal.pone.0120352 PMC4363671

[CR9] Bai Y, Zhang Y, Han B et al (2018) Circular RNA DLGAP4 Ameliorates Ischemic Stroke Outcomes by Targeting miR-143 to Regulate Endothelial-Mesenchymal Transition Associated with Blood–Brain Barrier Integrity. J Neurosci 38:32–50. 10.1523/JNEUROSCI.1348-17.2017 29114076 10.1523/JNEUROSCI.1348-17.2017 PMC6705810

[CR10] Barbagallo D, Palermo CI, Barbagallo C et al (2022) Competing endogenous RNA network mediated by circ_3205 in SARS-CoV-2 infected cells. Cell Mol Life Sci 79:75. 10.1007/s00018-021-04119-8 35039944 10.1007/s00018-021-04119-8 PMC8763136

[CR11] Bateman RJ, Aisen PS, De Strooper B et al (2010) Autosomal-dominant Alzheimer’s disease: a review and proposal for the prevention of Alzheimer’s disease. Alzheimers Res Ther 3:1. 10.1186/alzrt59 10.1186/alzrt59 PMC3109410 21211070

[CR12] Bentley DL (2014) Coupling mRNA processing with transcription in time and space. Nat Rev Genet 15:163–175. 10.1038/nrg3662 24514444 10.1038/nrg3662 PMC4304646

[CR13] Beric A, Sun Y, Sanchez S et al (2024) Circulating blood circular RNA in Parkinson’s Disease; from involvement in pathology to diagnostic tools in at-risk individuals. Npj Park Dis 10:222. 10.1038/s41531-024-00839-3 10.1038/s41531-024-00839-3 PMC11574145 39557914

[CR14] Beylerli O, Beilerli A, Ilyasova T et al (2024) CircRNAs in Alzheimer’s disease: What are the prospects? Non-Coding RNA Res 9:203–210. 10.1016/j.ncrna.2023.11.011 10.1016/j.ncrna.2023.11.011 PMC10730436 38125754

[CR15] Bhardwaj V, Singh A, Choudhary A et al (2023) HIV-1 Vpr induces ciTRAN to prevent transcriptional repression of the provirus. Sci Adv 9:eadh9170. 10.1126/sciadv.adh9170 37672576 10.1126/sciadv.adh9170 PMC10482341

[CR16] Bigarré IM, Trombetta BA, Guo Y et al (2021) I *GF2R* circular RNA hsa_circ_0131235 expression in the middle temporal cortex is associated with AD pathology. Brain Behav 11:e02048. 10.1002/brb3.2048 33704916 10.1002/brb3.2048 PMC8035435

[CR17] Bose R, Ain R (2018) Regulation of Transcription by Circular RNAs. In: Xiao J (ed) Circular RNAs. Springer Singapore, Singapore, pp 81–94 10.1007/978-981-13-1426-1_7 30259359

[CR18] Cao C, Zhang J, Zhang Z et al (2021) Knockdown circular RNA circGFRA1 inhibits glioma cell proliferation and migration by upregulating microRNA-99a. NeuroReport 32:748–756. 10.1097/WNR.0000000000001649 33994521 10.1097/WNR.0000000000001649

[CR19] Cao X, Guo J, Mochizuki H et al (2022) Circular RNA circ_0070441 regulates MPP+-triggered neurotoxic effect in SH-SY5Y cells via miR-626/IRS2 axis. Metab Brain Dis 37:513–524. 10.1007/s11011-021-00869-3 34748128 10.1007/s11011-021-00869-3

[CR20] Cervera-Carles L, Dols-Icardo O, Molina-Porcel L et al (2020) Assessing circular RNAs in Alzheimer’s disease and frontotemporal lobar degeneration. Neurobiol Aging 92:7–11. 10.1016/j.neurobiolaging.2020.03.017 32335360 10.1016/j.neurobiolaging.2020.03.017

[CR21] Chatoo W, Abdouh M, David J et al (2009) The Polycomb Group Gene *Bmi1* Regulates Antioxidant Defenses in Neurons by Repressing *p53* Pro-Oxidant Activity. J Neurosci 29:529–542. 10.1523/JNEUROSCI.5303-08.2009 19144853 10.1523/JNEUROSCI.5303-08.2009 PMC2744209

[CR23] Chen L-L (2016) The biogenesis and emerging roles of circular RNAs. Nat Rev Mol Cell Biol 17:205–211. 10.1038/nrm.2015.32 26908011 10.1038/nrm.2015.32

[CR22] Chen I, Chen C, Chuang T (2015) Biogenesis, identification, and function of exonic circular RNAs. WIREs RNA 6:563–579. 10.1002/wrna.1294 26230526 10.1002/wrna.1294 PMC5042038

[CR27] Chen YG, Kim MV, Chen X et al (2017) Sensing Self and Foreign Circular RNAs by Intron Identity. Mol Cell 67:228–238e5. 10.1016/j.molcel.2017.05.022 28625551 10.1016/j.molcel.2017.05.022 PMC5610545

[CR26] Chen YG, Chen R, Ahmad S et al (2019) N6-Methyladenosine Modification Controls Circular RNA Immunity. Mol Cell 76:96–109e9. 10.1016/j.molcel.2019.07.016 31474572 10.1016/j.molcel.2019.07.016 PMC6778039

[CR25] Chen W, Wang H, Zhu Z et al (2020) Exosome-Shuttled circSHOC2 from IPASs Regulates Neuronal Autophagy and Ameliorates Ischemic Brain Injury via the miR-7670-3p/SIRT1 Axis. Mol Ther Nucleic Acids 22:657–672. 10.1016/j.omtn.2020.09.027 33230464 10.1016/j.omtn.2020.09.027 PMC7581834

[CR24] Chen W, Hou C, Wang Y et al (2022) Circular RNA circTLK1 regulates dopaminergic neuron injury during Parkinson‘s disease by targeting miR-26a-5p/DAPK1. Neurosci Lett 782:136638. 10.1016/j.neulet.2022.136638 35447224 10.1016/j.neulet.2022.136638

[CR28] Cheng Q, Cao X, Xue L et al (2019) CircPRKCI-miR-545/589-E2F7 axis dysregulation mediates hydrogen peroxide-induced neuronal cell injury. Biochem Biophys Res Commun 514:428–435. 10.1016/j.bbrc.2019.04.131 31053300 10.1016/j.bbrc.2019.04.131

[CR29] Chouraki V, Seshadri S (2014) Genetics of Alzheimer’s Disease. In: Friedmann T, Dunlap JC, Goodwin SF (eds) Advances in Genetics, vol 87. Elsevier, pp 245–294. 10.1016/B978-0-12-800149-3.00005-6 10.1016/B978-0-12-800149-3.00005-6 25311924

[CR30] Conn SJ, Pillman KA, Toubia J et al (2015) The RNA Binding Protein Quaking Regulates Formation of circRNAs. Cell 160:1125–1134. 10.1016/j.cell.2015.02.014 25768908 10.1016/j.cell.2015.02.014

[CR31] D’Ambra E, Santini T, Vitiello E et al (2021) Circ-Hdgfrp3 shuttles along neurites and is trapped in aggregates formed by ALS-associated mutant FUS. iScience 24:103504. 10.1016/j.isci.2021.103504 34934923 10.1016/j.isci.2021.103504 PMC8661529

[CR32] Dattilo D, Di Timoteo G, Setti A et al (2023) The m6A reader YTHDC1 and the RNA helicase DDX5 control the production of rhabdomyosarcoma-enriched circRNAs. Nat Commun 14:1898. 10.1038/s41467-023-37578-7 37019933 10.1038/s41467-023-37578-7 PMC10076346

[CR33] Deng Y, Song H, Xiao Y et al (2022) High-Throughput Sequencing to Investigate lncRNA-circRNA-miRNA-mRNA Networks Underlying the Effects of Beta-Amyloid Peptide and Senescence on Astrocytes. Front Genet 13:868856. 10.3389/fgene.2022.868856 35646066 10.3389/fgene.2022.868856 PMC9133720

[CR34] Di Timoteo G, Dattilo D, Centrón-Broco A et al (2020) Modulation of circRNA Metabolism by m6A Modification. Cell Rep 31:107641. 10.1016/j.celrep.2020.107641 32402287 10.1016/j.celrep.2020.107641

[CR35] Dolinar A, Koritnik B, Glavač D, Ravnik-Glavač M (2019) Circular RNAs as Potential Blood Biomarkers in Amyotrophic Lateral Sclerosis. Mol Neurobiol 56:8052–8062. 10.1007/s12035-019-1627-x 31175544 10.1007/s12035-019-1627-x PMC6834740

[CR36] Dong X, Bai Y, Liao Z et al (2023) Circular RNAs in the human brain are tailored to neuron identity and neuropsychiatric disease. Nat Commun 14:5327. 10.1038/s41467-023-40348-0 37723137 10.1038/s41467-023-40348-0 PMC10507039

[CR40] Du WW, Yang W, Liu E et al (2016) Foxo3 circular RNA retards cell cycle progression via forming ternary complexes with p21 and CDK2. Nucleic Acids Res 44:2846–2858. 10.1093/nar/gkw027 26861625 10.1093/nar/gkw027 PMC4824104

[CR37] Du WW, Fang L, Yang W et al (2017) Induction of tumor apoptosis through a circular RNA enhancing Foxo3 activity. Cell Death Differ 24:357–370. 10.1038/cdd.2016.133 27886165 10.1038/cdd.2016.133 PMC5299715

[CR38] Du WW, Yang W, Li X et al (2018) A circular RNA circ-DNMT1 enhances breast cancer progression by activating autophagy. Oncogene 37:5829–5842. 10.1038/s41388-018-0369-y 29973691 10.1038/s41388-018-0369-y

[CR39] Du WW, Yang W, Li X et al (2020) The Circular RNA circSKA3 Binds Integrin β1 to Induce Invadopodium Formation Enhancing Breast Cancer Invasion. Mol Ther 28:1287–1298. 10.1016/j.ymthe.2020.03.002 32229309 10.1016/j.ymthe.2020.03.002 PMC7210749

[CR41] Dube U, Del-Aguila JL, Li Z et al (2019) An atlas of cortical circular RNA expression in Alzheimer disease brains demonstrates clinical and pathological associations. Nat Neurosci 22:1903–1912. 10.1038/s41593-019-0501-5 31591557 10.1038/s41593-019-0501-5 PMC6858549

[CR42] Elsayed LEO, Drouet V, Usenko T et al (2016) A N ovel N onsense M utation in *DNAJC 6* E xpands the P henotype of A utosomal- R ecessive J uvenile‐ O nset P arkinson’s D isease. Ann Neurol 79:335–337. 10.1002/ana.24591 26703368 10.1002/ana.24591

[CR43] Erdmann EA, Mahapatra A, Mukherjee P et al (2021) To protect and modify double-stranded RNA – the critical roles of ADARs in development, immunity and oncogenesis. Crit Rev Biochem Mol Biol 56:54–87. 10.1080/10409238.2020.1856768 33356612 10.1080/10409238.2020.1856768 PMC8019592

[CR44] Errichelli L, Dini Modigliani S, Laneve P et al (2017) FUS affects circular RNA expression in murine embryonic stem cell-derived motor neurons. Nat Commun 8:14741. 10.1038/ncomms14741 28358055 10.1038/ncomms14741 PMC5379105

[CR45] Feng Z, Zhang L, Wang S, Hong Q (2020) Circular RNA circDLGAP4 exerts neuroprotective effects via modulating miR-134-5p/CREB pathway in Parkinson’s disease. Biochem Biophys Res Commun 522:388–394. 10.1016/j.bbrc.2019.11.102 31761328 10.1016/j.bbrc.2019.11.102

[CR46] Fischer JW, Busa VF, Shao Y, Leung AKL (2020) Structure-Mediated RNA Decay by UPF1 and G3BP1. Mol Cell 78:70–84e6. 10.1016/j.molcel.2020.01.021 32017897 10.1016/j.molcel.2020.01.021 PMC8055448

[CR47] Ghafouri-Fard S, Safari M, Taheri M, Samadian M (2022) Expression of Linear and Circular lncRNAs in Alzheimer’s Disease. J Mol Neurosci 72:187–200. 10.1007/s12031-021-01900-z 34415549 10.1007/s12031-021-01900-z

[CR48] Gou F, Gao Y, Zhong K et al (2025) Roles and Applications of Circular RNA in Virus Infection. Int J Mol Sci 26:9656. 10.3390/ijms26199656 41096921 10.3390/ijms26199656 PMC12524399

[CR49] Greenough MA (2016) The Role of Presenilin in Protein Trafficking and Degradation—Implications for Metal Homeostasis. J Mol Neurosci 60:289–297. 10.1007/s12031-016-0826-4 27558108 10.1007/s12031-016-0826-4

[CR50] Gruner H, Cortés-López M, Cooper DA et al (2016) CircRNA accumulation in the aging mouse brain. Sci Rep 6:38907. 10.1038/srep38907 27958329 10.1038/srep38907 PMC5153657

[CR176] Hales CM, Rees H, Seyfried NT et al (2013) Abnormal gephyrin immunoreactivity associated with Alzheimer disease pathologic changes. J Neuropathol Exp Neurol 72:1009–1015. 10.1097/01.jnen.0000435847.59828.db 24128675 10.1097/01.jnen.0000435847.59828.db PMC4037931

[CR51] Han B, Zhang Y, Zhang Y et al (2018) Novel insight into circular RNA *HECTD1* in astrocyte activation via autophagy by targeting *MIR142* -TIPARP: implications for cerebral ischemic stroke. Autophagy 14:1164–1184. 10.1080/15548627.2018.1458173 29938598 10.1080/15548627.2018.1458173 PMC6103660

[CR52] Hanan M, Simchovitz A, Yayon N et al (2020) A Parkinson’s disease Circ RNA s Resource reveals a link between circ SLC 8A1 and oxidative stress. EMBO Mol Med 12:e11942. 10.15252/emmm.201911942 32715657 10.15252/emmm.201911942 PMC7507321

[CR55] Hébert SS, Horré K, Nicolaï L et al (2008) Loss of microRNA cluster miR-29a/b-1 in sporadic Alzheimer’s disease correlates with increased BACE1/β-secretase expression. Proc Natl Acad Sci 105:6415–6420. 10.1073/pnas.0710263105 18434550 10.1073/pnas.0710263105 PMC2359789

[CR54] He W, Auclert LZ, Zhai X et al (2019) Interspecies Transmission, Genetic Diversity, and Evolutionary Dynamics of Pseudorabies Virus. J Infect Dis 219:1705–1715. 10.1093/infdis/jiy731 30590733 10.1093/infdis/jiy731

[CR53] He L, Zhang F, Zhu Y, Lu M (2022) A crosstalk between circular RNA, microRNA, and messenger RNA in the development of various brain cognitive disorders. Front Mol Neurosci 15:960657. 10.3389/fnmol.2022.960657 36329693 10.3389/fnmol.2022.960657 PMC9622787

[CR56] Hong X, Liu N, Liang Y et al (2020) Circular RNA CRIM1 functions as a ceRNA to promote nasopharyngeal carcinoma metastasis and docetaxel chemoresistance through upregulating FOXQ1. Mol Cancer 19:33. 10.1186/s12943-020-01149-x 32061262 10.1186/s12943-020-01149-x PMC7023763

[CR57] Hu Y, Yu Y, Yang R et al (2023) The neuropathological mechanism of EV-A71 infection attributes to inflammatory pryoptosis and viral replication via activating the hsa_circ_0045431/ hsa_miR_584/NLRP3 regulatory axis. Virus Res 335:199195. 10.1016/j.virusres.2023.199195 37579846 10.1016/j.virusres.2023.199195 PMC10450994

[CR61] Huang Y-WA, Zhou B, Wernig M, Südhof TC (2017) ApoE2, ApoE3, and ApoE4 Differentially Stimulate APP Transcription and Aβ Secretion. Cell 168:427–441e21. 10.1016/j.cell.2016.12.044 28111074 10.1016/j.cell.2016.12.044 PMC5310835

[CR58] Huang J-L, Qin M-C, Zhou Y et al (2018a) Comprehensive analysis of differentially expressed profiles of Alzheimer’s disease associated circular RNAs in an Alzheimer’s disease mouse model. Aging 10:253–265. 10.18632/aging.101387 29448241 10.18632/aging.101387 PMC5842852

[CR60] Huang J-L, Xu Z-H, Yang S-M et al (2018b) Identification of Differentially Expressed Profiles of Alzheimer’s Disease Associated Circular RNAs in a Panax Notoginseng Saponins-Treated Alzheimer’s Disease Mouse Model. Comput Struct Biotechnol J 16:523–531. 10.1016/j.csbj.2018.10.010 30524667 10.1016/j.csbj.2018.10.010 PMC6260282

[CR59] Huang J-L, Su M, Wu D-P (2020) Functional roles of circular RNAs in Alzheimer’s disease. Ageing Res Rev 60:101058. 10.1016/j.arr.2020.101058 32234545 10.1016/j.arr.2020.101058

[CR62] Ivanov A, Memczak S, Wyler E et al (2015) Analysis of Intron Sequences Reveals Hallmarks of Circular RNA Biogenesis in Animals. Cell Rep 10:170–177. 10.1016/j.celrep.2014.12.019 25558066 10.1016/j.celrep.2014.12.019

[CR64] Jia J, Xu J, Liu J et al (2021) Comprehensive Management of Daily Living Activities, behavioral and Psychological Symptoms, and Cognitive Function in Patients with Alzheimer’s Disease: A Chinese Consensus on the Comprehensive Management of Alzheimer’s Disease. Neurosci Bull 37:1025–1038. 10.1007/s12264-021-00701-z 34050523 10.1007/s12264-021-00701-z PMC8275730

[CR65] Jiang W, Wang L, Zhang Y, Li H (2020) Circ-ATP5H Induces Hepatitis B Virus Replication and Expression by Regulating miR-138-5p/TNFAIP3 Axis. Cancer Manag Res Volume 12:11031–11040. 10.2147/CMAR.S272983 10.2147/CMAR.S272983 PMC7648158 33173336

[CR66] Jost I, Shalamova LA, Gerresheim GK et al (2018) Functional sequestration of microRNA-122 from Hepatitis C Virus by circular RNA sponges. RNA Biol 1–8. 10.1080/15476286.2018.1435248 10.1080/15476286.2018.1435248 PMC6161685 29486652

[CR177] KangL, Xie H, Ye H et al (2023) Hsa_circ_0007321 regulates Zika virus replication through miR-492/NFKBID/NF-κB signaling pathway. J Virol 97:e01232–e01223. 10.1128/jvi.01232-23 38051045 10.1128/jvi.01232-23 PMC10734422

[CR67] Katsumoto A, Takeuchi H, Takahashi K, Tanaka F (2018) Microglia in Alzheimer’s Disease: Risk Factors and Inflammation. Front Neurol 9:978. 10.3389/fneur.2018.00978 30498474 10.3389/fneur.2018.00978 PMC6249341

[CR68] Kerr F, Sofola-Adesakin O, Ivanov DK et al (2017) Direct Keap1-Nrf2 disruption as a potential therapeutic target for Alzheimer’s disease. PLOS Genet 13:e1006593. 10.1371/journal.pgen.1006593 28253260 10.1371/journal.pgen.1006593 PMC5333801

[CR69] Kim G, Gautier O, Tassoni-Tsuchida E et al (2020) ALS Genetics: Gains, Losses, and Implications for Future Therapies. Neuron 108:822–842. 10.1016/j.neuron.2020.08.022 32931756 10.1016/j.neuron.2020.08.022 PMC7736125

[CR70] Kristensen LS, Andersen MS, Stagsted LVW et al (2019) The biogenesis, biology and characterization of circular RNAs. Nat Rev Genet 20:675–691. 10.1038/s41576-019-0158-7 31395983 10.1038/s41576-019-0158-7

[CR71] Krokidis MG, Exarchos TP, Vlamos P, Bioinformatics, Laboratory HE, Department of Informatics, University I, Greece (2021) Data-driven biomarker analysis using computational omics approaches to assess neurodegenerative disease progression. Math Biosci Eng 18:1813–1832. 10.3934/mbe.2021094 33757212 10.3934/mbe.2021094

[CR72] Kumar L, Shamsuzzama, Jadiya P et al (2018) Functional Characterization of Novel Circular RNA Molecule, circzip-2 and Its Synthesizing Gene zip-2 in C. elegans Model of Parkinson’s Disease. Mol Neurobiol 55:6914–6926. 10.1007/s12035-018-0903-5 29363043 10.1007/s12035-018-0903-5

[CR73] Lane CA, Hardy J, Schott JM (2018) Alzheimer’s disease. Eur J Neurol 25:59–70. 10.1111/ene.13439 28872215 10.1111/ene.13439

[CR74] Legnini I, Di Timoteo G, Rossi F et al (2017) Circ-ZNF609 Is a Circular RNA that Can Be Translated and Functions in Myogenesis. Mol Cell 66:22–37e9. 10.1016/j.molcel.2017.02.017 28344082 10.1016/j.molcel.2017.02.017 PMC5387670

[CR78] Li X, Liu C-X, Xue W et al (2017) Coordinated circRNA Biogenesis and Function with NF90/NF110 in Viral Infection. Mol Cell 67:214–227e7. 10.1016/j.molcel.2017.05.023 28625552 10.1016/j.molcel.2017.05.023

[CR81] LiY, Fan H, Sun J et al (2020b) Circular RNA expression profile of Alzheimer’s disease and its clinical significance as biomarkers for the disease risk and progression. Int J Biochem Cell Biol 123:105747. 10.1016/j.biocel.2020.105747 10.1016/j.biocel.2020.105747 32315771

[CR80] Li Y, Ashraf U, Chen Z et al (2020a) Genome-wide profiling of host-encoded circular RNAs highlights their potential role during the Japanese encephalitis virus-induced neuroinflammatory response. BMC Genomics 21:409. 10.1186/s12864-020-06822-5 32552669 10.1186/s12864-020-06822-5 PMC7301528

[CR82] Li Y, Lv Z, Zhang J et al (2020c) Profiling of differentially expressed circular RNAs in peripheral blood mononuclear cells from Alzheimer’s disease patients. Metab Brain Dis 35:201–213. 10.1007/s11011-019-00497-y 31834549 10.1007/s11011-019-00497-y

[CR76] Li M, Hu J, Peng Y et al (2021) CircPTK2-miR-181c-5p-HMGB1: a new regulatory pathway for microglia activation and hippocampal neuronal apoptosis induced by sepsis. Mol Med 27:45. 10.1186/s10020-021-00305-3 33952191 10.1186/s10020-021-00305-3 PMC8101146

[CR77] Li N, Zhang D, Guo H et al (2022a) Inhibition of circ_0004381 improves cognitive function via miR-647/PSEN1 axis in an Alzheimer disease mouse model. J Neuropathol Exp Neurol 82:84–92. 10.1093/jnen/nlac108 36409993 10.1093/jnen/nlac108

[CR83] Li Y, Wang H, Chen L et al (2022b) Circ_0003611 regulates apoptosis and oxidative stress injury of Alzheimer’s disease via miR-383-5p/KIF1B axis. Metab Brain Dis 37:2915–2924. 10.1007/s11011-022-01051-z 35960460 10.1007/s11011-022-01051-z

[CR172] Li Y, Wang H, Chen L et al (2022c) Circ_0003611 regulates apoptosis and oxidative stress injury of Alzheimer’s disease via miR-383-5p/KIF1B axis. Metab Brain Dis 37:2915–2924. 10.1007/s11011-022-01051-z 10.1007/s11011-022-01051-z 35960460

[CR75] Li H, Du L, Li J et al (2024a) A previously unidentified circRNA inhibits virus replication by regulating the miR-24-3p/KEAP1 axis. PLOS Pathog 20:e1012712. 10.1371/journal.ppat.1012712 39689152 10.1371/journal.ppat.1012712 PMC11651552

[CR79] Li X, Wang P, Qi S et al (2024b) The clinical perspective of circular RNAs in neurodegenerative diseases: potential diagnostic tools and therapeutic targets. Front Cell Neurosci 18:1470641. 10.3389/fncel.2024.1470641 39624645 10.3389/fncel.2024.1470641 PMC11608961

[CR84] Liang D, Wilusz JE (2014) Short intronic repeat sequences facilitate circular RNA production. Genes Dev 28:2233–2247. 10.1101/gad.251926.114 25281217 10.1101/gad.251926.114 PMC4201285

[CR85] Liao R, Liu L, Zhou J et al (2021) Current Molecular Biology and Therapeutic Strategy Status and Prospects for circRNAs in HBV-Associated Hepatocellular Carcinoma. Front Oncol 11:697747. 10.3389/fonc.2021.697747 34277444 10.3389/fonc.2021.697747 PMC8284075

[CR87] Liu L, Chen X, Chen Y-H, Zhang K (2020) Identification of Circular RNA hsa_Circ_0003391 in Peripheral Blood Is Potentially Associated With Alzheimer’s Disease. Front Aging Neurosci 12:601965. 10.3389/fnagi.2020.601965 33424579 10.3389/fnagi.2020.601965 PMC7793744

[CR86] Liu G, Peng J, Liao Z et al (2021) Genome-wide survival study identifies a novel synaptic locus and polygenic score for cognitive progression in Parkinson’s disease. Nat Genet 53:787–793. 10.1038/s41588-021-00847-6 33958783 10.1038/s41588-021-00847-6 PMC8459648

[CR88] Liu Q, Li Q, Zhang R et al (2022a) circ-Pank1 promotes dopaminergic neuron neurodegeneration through modulating miR-7a-5p/α-syn pathway in Parkinson’s disease. Cell Death Dis 13:477. 10.1038/s41419-022-04934-2 35589691 10.1038/s41419-022-04934-2 PMC9120029

[CR89] Liu X, Zhang Y, Zhou S et al (2022b) Circular RNA: An emerging frontier in RNA therapeutic targets, RNA therapeutics, and mRNA vaccines. J Controlled Release 348:84–94. 10.1016/j.jconrel.2022.05.043 10.1016/j.jconrel.2022.05.043 PMC9644292 35649485

[CR90] Lo Ij, Hill J, Vilhjálmsson BJ, Kjems J (2020) Linking the association between circRNAs and Alzheimer’s disease progression by multi-tissue circular RNA characterization. RNA Biol 17:1789–1797. 10.1080/15476286.2020.1783487 32618510 10.1080/15476286.2020.1783487 PMC7714474

[CR91] Long JM, Lahiri DK (2011) MicroRNA-101 downregulates Alzheimer’s amyloid-β precursor protein levels in human cell cultures and is differentially expressed. Biochem Biophys Res Commun 404:889–895. 10.1016/j.bbrc.2010.12.053 21172309 10.1016/j.bbrc.2010.12.053 PMC3372402

[CR92] Lu Y, Tan L, Wang X (2019) Circular HDAC9/microRNA-138/Sirtuin-1 Pathway Mediates Synaptic and Amyloid Precursor Protein Processing Deficits in Alzheimer’s Disease. Neurosci Bull 35:877–888. 10.1007/s12264-019-00361-0 30887246 10.1007/s12264-019-00361-0 PMC6754481

[CR93] Ma M, Xie D, Zhao J (2023) Bioinformatics and Experimental Identification of circ_0001535 Associated with Diagnosis and Development of Alzheimer’s Disease. J Integr Neurosci 22:105. 10.31083/j.jin2204105 37519165 10.31083/j.jin2204105

[CR94] Mahmoudi E, Cairns MJ (2019) Circular RNAs are temporospatially regulated throughout development and ageing in the rat. Sci Rep 9:2564. 10.1038/s41598-019-38860-9 30796328 10.1038/s41598-019-38860-9 PMC6385508

[CR95] Mecozzi N, Nenci A, Vera O et al (2022) Genetic tools for the stable overexpression of circular RNAs. RNA Biol 19:353–363. 10.1080/15476286.2022.2043041 35289721 10.1080/15476286.2022.2043041 PMC8928841

[CR96] Memczak S, Jens M, Elefsinioti A et al (2013) Circular RNAs are a large class of animal RNAs with regulatory potency. Nature 495:333–338. 10.1038/nature11928 23446348 10.1038/nature11928

[CR97] Meng S, Zhou H, Feng Z et al (2017) CircRNA: functions and properties of a novel potential biomarker for cancer. Mol Cancer 16:94. 10.1186/s12943-017-0663-2 28535767 10.1186/s12943-017-0663-2 PMC5440908

[CR98] Millan MJ (2017) Linking deregulation of non-coding RNA to the core pathophysiology of Alzheimer’s disease: An integrative review. Prog Neurobiol 156:1–68. 10.1016/j.pneurobio.2017.03.004 28322921 10.1016/j.pneurobio.2017.03.004

[CR100] Min S-W, Sohn PD, Cho S-H et al (2013) Sirtuins in neurodegenerative diseases: an update on potential mechanisms. Front Aging Neurosci 5. 10.3389/fnagi.2013.00053 10.3389/fnagi.2013.00053 PMC3782645 24093018

[CR99] Min J, Li Y, Li X et al (2023) The circRNA circVAMP3 restricts influenza A virus replication by interfering with NP and NS1 proteins. PLOS Pathog 19:e1011577. 10.1371/journal.ppat.1011577 37603540 10.1371/journal.ppat.1011577 PMC10441791

[CR101] Mo D, Li X, Raabe CA et al (2020) Circular RNA Encoded Amyloid Beta peptides—A Novel Putative Player in Alzheimer’s Disease. Cells 9:2196. 10.3390/cells9102196 33003364 10.3390/cells9102196 PMC7650678

[CR102] Nalls MA, Blauwendraat C, Vallerga CL et al (2019) Identification of novel risk loci, causal insights, and heritable risk for Parkinson’s disease: a meta-analysis of genome-wide association studies. Lancet Neurol 18:1091–1102. 10.1016/S1474-4422(19)30320-5 31701892 10.1016/S1474-4422(19)30320-5 PMC8422160

[CR103] Olgiati S, Quadri M, Fang M et al (2016) *D NAJC 6* M utations A ssociated W ith E arly- O nset P arkinson’s D isease. Ann Neurol 79:244–256. 10.1002/ana.24553 26528954 10.1002/ana.24553

[CR104] Pan Z, Li G-F, Sun M-L et al (2019) MicroRNA-1224 Splicing CircularRNA-Filip1l in an Ago2-Dependent Manner Regulates Chronic Inflammatory Pain via Targeting Ubr5. J Neurosci 39:2125–2143. 10.1523/JNEUROSCI.1631-18.2018 30651325 10.1523/JNEUROSCI.1631-18.2018 PMC6507086

[CR173] Pan W, Hu Y, Wang L, Li J (2022) Circ_0003611 acts as a miR-885-5p sponge to aggravate the amyloid-β-induced neuronal injury in Alzheimer’s disease. Metab Brain Dis 37:961–971. 10.1007/s11011-022-00912-x 35076819 10.1007/s11011-022-00912-x

[CR105] Pereira PA, Tomás JF, Queiroz JA et al (2016) Recombinant pre-miR-29b for Alzheimer´s disease therapeutics. Sci Rep 6:19946. 10.1038/srep19946 26818210 10.1038/srep19946 PMC4730146

[CR106] Pierouli K, Papakonstantinou E, Papageorgiou L et al (2022) Role of non–coding RNAs as biomarkers and the application of omics technologies in Alzheimer’s disease (Review). Int J Mol Med 51:5. 10.3892/ijmm.2022.5208 36453246 10.3892/ijmm.2022.5208 PMC9747195

[CR107] Pisignano G, Michael DC, Visal TH et al (2023) Going circular: history, present, and future of circRNAs in cancer. Oncogene 42:2783–2800. 10.1038/s41388-023-02780-w 37587333 10.1038/s41388-023-02780-w PMC10504067

[CR108] Piwecka M, Glažar P, Hernandez-Miranda LR et al (2017) Loss of a mammalian circular RNA locus causes miRNA deregulation and affects brain function. Science 357:eaam8526. 10.1126/science.aam8526 28798046 10.1126/science.aam8526

[CR109] Qiu H, Yang B, Chen Y et al (2023) Influenza A Virus-Induced circRNA circMerTK Negatively Regulates Innate Antiviral Responses. Microbiol Spectr 11:e03637–e03622. 10.1128/spectrum.03637-22 36847523 10.1128/spectrum.03637-22 PMC10100971

[CR111] Qu S, Yang X, Li X et al (2015) Circular RNA: A new star of noncoding RNAs. Cancer Lett 365:141–148. 10.1016/j.canlet.2015.06.003 26052092 10.1016/j.canlet.2015.06.003

[CR110] Qu L, Yi Z, Shen Y et al (2022) Circular RNA vaccines against SARS-CoV-2 and emerging variants. Cell 185:1728–1744e16. 10.1016/j.cell.2022.03.044 35460644 10.1016/j.cell.2022.03.044 PMC8971115

[CR112] Ravanidis S, Bougea A, Karampatsi D et al (2021) Differentially Expressed Circular RNAs in Peripheral Blood Mononuclear Cells of Patients with Parkinson’s Disease. Mov Disord 36:1170–1179. 10.1002/mds.28467 33433033 10.1002/mds.28467 PMC8248110

[CR113] Reinhold D, Farztdinov V, Yan Y et al (2023) The brain reacting to COVID-19: analysis of the cerebrospinal fluid proteome, RNA and inflammation. J Neuroinflammation 20:30. 10.1186/s12974-023-02711-2 36759861 10.1186/s12974-023-02711-2 PMC9909638

[CR63] Saadh J, Abedi Kiasari M, Shahrtash B SA, et al (2023) Exosomal non-coding RNAs’ role in immune regulation and potential therapeutic applications. Pathol - Res Pract 247:154522. 10.1016/j.prp.2023.154522 37201467 10.1016/j.prp.2023.154522

[CR114] Sang Q, Liu X, Wang L et al (2018) CircSNCA downregulation by pramipexole treatment mediates cell apoptosis and autophagy in Parkinson’s disease by targeting miR-7. Aging 10:1281–1293. 10.18632/aging.101466 29953413 10.18632/aging.101466 PMC6046232

[CR115] Saunders AM, Strittmatter WJ, Schmechel D et al (1993) Association of apolipoprotein E allele ϵ4 with late-onset familial and sporadic Alzheimer’s disease. Neurology 43:1467–1467. 10.1212/WNL.43.8.1467 8350998 10.1212/wnl.43.8.1467

[CR116] Shafik AM, Zhang F, Guo Z et al (2021) N6-methyladenosine dynamics in neurodevelopment and aging, and its potential role in Alzheimer’s disease. Genome Biol 22:17. 10.1186/s13059-020-02249-z 33402207 10.1186/s13059-020-02249-z PMC7786910

[CR117] Sharma AR, Bhattacharya M, Bhakta S et al (2021) Recent research progress on circular RNAs: Biogenesis, properties, functions, and therapeutic potential. Mol Ther - Nucleic Acids 25:355–371. 10.1016/j.omtn.2021.05.022 34484862 10.1016/j.omtn.2021.05.022 PMC8399087

[CR119] Shen T, Han M, Wei G, Ni T (2015) An intriguing RNA species—perspectives of circularized RNA. Protein Cell 6:871–880. 10.1007/s13238-015-0202-0 26349458 10.1007/s13238-015-0202-0 PMC4656206

[CR118] Shen H, An O, Ren X et al (2022) ADARs act as potent regulators of circular transcriptome in cancer. Nat Commun 13:1508. 10.1038/s41467-022-29138-2 35314703 10.1038/s41467-022-29138-2 PMC8938519

[CR120] Shi Z, Chen T, Yao Q et al (2017) The circular RNA ci RS -7 promotes APP and BACE 1 degradation in an NF ‐κB‐dependent manner. FEBS J 284:1096–1109. 10.1111/febs.14045 28296235 10.1111/febs.14045

[CR121] Smeyne M, Smeyne RJ (2013) Glutathione metabolism and Parkinson’s disease. Free Radic Biol Med 62:13–25. 10.1016/j.freeradbiomed.2013.05.001 23665395 10.1016/j.freeradbiomed.2013.05.001 PMC3736736

[CR175] SmolinskyB, Eichler SA, Buchmeier S et al (2008) Splice-specific functions of gephyrin in molybdenum cofactor biosynthesis. J Biol Chem 283:17370–17379. 10.1074/jbc.M800985200 18411266 10.1074/jbc.M800985200

[CR123] Song Q, Peng S, Zhu X (2021) Baicalein protects against MPP+/MPTP-induced neurotoxicity by ameliorating oxidative stress in SH-SY5Y cells and mouse model of Parkinson’s disease. Neurotoxicology 87:188–194. 10.1016/j.neuro.2021.10.003 34666128 10.1016/j.neuro.2021.10.003

[CR122] Song C, Zhang Y, Huang W et al (2022) Circular RNA Cwc27 contributes to Alzheimer’s disease pathogenesis by repressing Pur-α activity. Cell Death Differ 29:393–406. 10.1038/s41418-021-00865-1 34504314 10.1038/s41418-021-00865-1 PMC8817017

[CR124] Tagawa T, Oh D, Dremel S et al (2023) A virus-induced circular RNA maintains latent infection of Kaposi’s sarcoma herpesvirus. Proc Natl Acad Sci 120:e2212864120. 10.1073/pnas.2212864120 36724259 10.1073/pnas.2212864120 PMC9963958

[CR125] Titze-de-Almeida SS, Titze-de-Almeida R (2023) Progress in circRNA-targeted therapy in experimental Parkinson’s disease. Pharmaceutics 15:2035. 10.3390/pharmaceutics15082035 37631249 10.3390/pharmaceutics15082035 PMC10459713

[CR126] Urdánoz-Casado A, Sánchez-Ruiz De Gordoa J, Robles M et al (2021) Gender-Dependent Deregulation of Linear and Circular RNA Variants of HOMER1 in the Entorhinal Cortex of Alzheimer’s Disease. Int J Mol Sci 22:9205. 10.3390/ijms22179205 34502114 10.3390/ijms22179205 PMC8430762

[CR127] Urdánoz-Casado A, Sánchez-Ruiz De Gordoa J, Robles M et al (2023) circRNA from APP Gene Changes in Alzheimer’s Disease Human Brain. Int J Mol Sci 24:4308. 10.3390/ijms24054308 36901741 10.3390/ijms24054308 PMC10002054

[CR128] Valizadeh M, Derafsh E, Abdi Abyaneh F et al (2024) Non-Coding RNAs and Neurodegenerative Diseases: Information of their Roles in Apoptosis. Mol Neurobiol 61:4508–4537. 10.1007/s12035-023-03849-z 38102518 10.1007/s12035-023-03849-z

[CR134] Wang Y, Wang Z (2015) Efficient backsplicing produces translatable circular mRNAs. RNA 21:172–179. 10.1261/rna.048272.114 25449546 10.1261/rna.048272.114 PMC4338345

[CR130] Wang PL, Bao Y, Yee M-C et al (2014) Circular RNA Is Expressed across the Eukaryotic Tree of Life. PLoS ONE 9:e90859. 10.1371/journal.pone.0090859 24609083 10.1371/journal.pone.0090859 PMC3946582

[CR133] Wang X, Tan L, Lu Y et al (2015) MicroRNA-138 promotes tau phosphorylation by targeting retinoic acid receptor alpha. FEBS Lett 589:726–729. 10.1016/j.febslet.2015.02.001 25680531 10.1016/j.febslet.2015.02.001

[CR129] Wang H, Li Z, Gao J, Liao Q (2019) Circular RNA circPTK2 regulates oxygen-glucose deprivation-activated microglia-induced hippocampal neuronal apoptosis via miR-29b-SOCS-1-JAK2/STAT3-IL-1β signaling. Int J Biol Macromol 129:488–496. 10.1016/j.ijbiomac.2019.02.041 30742923 10.1016/j.ijbiomac.2019.02.041

[CR132] Wang W, Lv R, Zhang J, Liu Y (2021) circSAMD4A participates in the apoptosis and autophagy of dopaminergic neurons via the miR–29c–3p–mediated AMPK/mTOR pathway in Parkinson’s disease. Mol Med Rep 24:540. 10.3892/mmr.2021.12179 34080649 10.3892/mmr.2021.12179 PMC8170871

[CR131] Wang RC, Lee EE, Zhao J, Kim J (2022) Assessment of the Abundance and Potential Function of Human Papillomavirus Type 16 Circular E7 RNA. mBio 13:e00411–e00422. 10.1128/mbio.00411-22 35579354 10.1128/mbio.00411-22 PMC9239243

[CR174] Weinger JG, Brosnan CF, Loudig O et al (2011) Loss of the receptor tyrosine kinase Axl leads to enhanced inflammation in the CNS and delayed removal of myelin debris during Experimental Autoimmune Encephalomyelitis. J Neuroinflammation 8:49. 10.1186/1742-2094-8-49 21569627 10.1186/1742-2094-8-49 PMC3121615

[CR135] Welden JR, Van Doorn J, Nelson PT, Stamm S (2018) The human MAPT locus generates circular RNAs. Biochim Biophys Acta BBA - Mol Basis Dis 1864:2753–2760. 10.1016/j.bbadis.2018.04.023 10.1016/j.bbadis.2018.04.023 PMC6434521 29729314

[CR136] Wesselhoeft RA, Kowalski PS, Anderson DG (2018) Engineering circular RNA for potent and stable translation in eukaryotic cells. Nat Commun 9:2629. 10.1038/s41467-018-05096-6 29980667 10.1038/s41467-018-05096-6 PMC6035260

[CR137] Wesselhoeft RA, Kowalski PS, Parker-Hale FC et al (2019) RNA Circularization Diminishes Immunogenicity and Can Extend Translation Duration In Vivo. Mol Cell 74:508–520e4. 10.1016/j.molcel.2019.02.015 30902547 10.1016/j.molcel.2019.02.015 PMC6724735

[CR138] Whittle BJ, Izuogu OG, Lowes H et al (2024) Early-stage idiopathic Parkinson’s disease is associated with reduced circular RNA expression. Npj Park Dis 10:25. 10.1038/s41531-024-00636-y 10.1038/s41531-024-00636-y PMC10799891 38245550

[CR171] Wu L, Du Q, Wu C (2021) CircLPAR1/miR-212-3p/ZNF217 feedback loop promotes amyloid β-induced neuronal injury in Alzheimer’s Disease. Brain Res 1770:147622. 10.1016/j.brainres.2021.147622 34403662 10.1016/j.brainres.2021.147622

[CR139] Wu D, Zhao Y, Yan Q et al (2023) Circular RNAs: emerging players in brain aging and neurodegenerative diseases. J Pathol 259:1–9. 10.1002/path.6021 36264226 10.1002/path.6021

[CR140] Xiao F, He Z, Wang S et al (2024) Regulatory mechanism of circular RNAs in neurodegenerative diseases. CNS Neurosci Ther 30:e14499. 10.1111/cns.14499 37864389 10.1111/cns.14499 PMC11017410

[CR141] Xie Y, Zhang S, Lv Z et al (2021) SOX21-AS1 modulates neuronal injury of MMP+-treated SH-SY5Y cells via targeting miR-7-5p and inhibiting IRS2. Neurosci Lett 746:135602. 10.1016/j.neulet.2020.135602 33421490 10.1016/j.neulet.2020.135602

[CR142] Xu K, Zhang Y, Xiong W et al (2020) CircGRIA1 shows an age-related increase in male macaque brain and regulates synaptic plasticity and synaptogenesis. Nat Commun 11:3594. 10.1038/s41467-020-17435-7 32681011 10.1038/s41467-020-17435-7 PMC7367861

[CR143] Yan L, Chen YG (2020) Circular RNAs in Immune Response and Viral Infection. Trends Biochem Sci 45:1022–1034. 10.1016/j.tibs.2020.08.006 32900574 10.1016/j.tibs.2020.08.006 PMC7642119

[CR148] Yang Y, Fan X, Mao M et al (2017) Extensive translation of circular RNAs driven by N6-methyladenosine. Cell Res 27:626–641. 10.1038/cr.2017.31 28281539 10.1038/cr.2017.31 PMC5520850

[CR146] Yang J-H, Zhang R-J, Lin J-J et al (2018a) The Differentially Expressed Circular RNAs in the Substantia Nigra and Corpus Striatum of Nrf2-Knockout Mice. Cell Physiol Biochem 50:936–951. 10.1159/000494478 30355941 10.1159/000494478

[CR149] Yang Y, Gao X, Zhang M et al (2018b) Novel Role of FBXW7 Circular RNA in Repressing Glioma Tumorigenesis. JNCI J Natl Cancer Inst 110:304–315. 10.1093/jnci/djx166 28903484 10.1093/jnci/djx166 PMC6019044

[CR144] Yang F, Hu A, Li D et al (2019a) Circ-HuR suppresses HuR expression and gastric cancer progression by inhibiting CNBP transactivation. Mol Cancer 18:158. 10.1186/s12943-019-1094-z 31718709 10.1186/s12943-019-1094-z PMC6852727

[CR145] Yang H, Wang H, Shang H et al (2019b) Circular RNA circ_0000950 promotes neuron apoptosis, suppresses neurite outgrowth and elevates inflammatory cytokines levels via directly sponging miR-103 in Alzheimer’s disease. Cell Cycle 18:2197–2214. 10.1080/15384101.2019.1629773 31373242 10.1080/15384101.2019.1629773 PMC6738533

[CR147] Yang L, Han B, Zhang Z et al (2020) Extracellular Vesicle–Mediated Delivery of Circular RNA SCMH1 Promotes Functional Recovery in Rodent and Nonhuman Primate Ischemic Stroke Models. Circulation 142:556–574. 10.1161/CIRCULATIONAHA.120.045765 32441115 10.1161/CIRCULATIONAHA.120.045765

[CR150] Yin X, Li H, Zhou Y (2024) Circular RNAs in viral infection and antiviral treatment. Cells 13:2033. 10.3390/cells13232033 39682781 10.3390/cells13232033 PMC11640649

[CR151] You X, Vlatkovic I, Babic A et al (2015) Neural circular RNAs are derived from synaptic genes and regulated by development and plasticity. Nat Neurosci 18:603–610. 10.1038/nn.3975 25714049 10.1038/nn.3975 PMC4376664

[CR152] Yu X, Bai Y, Han B et al (2022) Extracellular vesicle-mediated delivery of circDYM alleviates CUS‐induced depressive‐like behaviours. J Extracell Vesicles 11:e12185. 10.1002/jev2.12185 35029057 10.1002/jev2.12185 PMC8758833

[CR153] Yue X, Zhong C, Cao R et al (2024) CircRNA based multivalent neuraminidase vaccine induces broad protection against influenza viruses in mice. Npj Vaccines 9:170. 10.1038/s41541-024-00963-4 39285168 10.1038/s41541-024-00963-4 PMC11405689

[CR157] Zhang L, Wang Z (2020) Circular RNA hsa_circ_0004812 impairs IFN-induced immune response by sponging miR-1287-5p to regulate FSTL1 in chronic hepatitis B. Virol J 17:40. 10.1186/s12985-020-01314-0 32188476 10.1186/s12985-020-01314-0 PMC7079541

[CR161] Zhang Y, Zhang X-O, Chen T et al (2013) Circular Intronic Long Noncoding RNAs. Mol Cell 51:792–806. 10.1016/j.molcel.2013.08.017 24035497 10.1016/j.molcel.2013.08.017

[CR159] Zhang X-O, Wang H-B, Zhang Y et al (2014) Complementary Sequence-Mediated Exon Circularization. Cell 159:134–147. 10.1016/j.cell.2014.09.001 25242744 10.1016/j.cell.2014.09.001

[CR160] Zhang Y, Yu F, Bao S, Sun J (2019) Systematic Characterization of Circular RNA-Associated CeRNA Network Identified Novel circRNA Biomarkers in Alzheimer’s Disease. Front Bioeng Biotechnol 7:222. 10.3389/fbioe.2019.00222 31572720 10.3389/fbioe.2019.00222 PMC6749152

[CR156] Zhang L, Hou C, Chen C et al (2020a) The role of N6-methyladenosine (m6A) modification in the regulation of circRNAs. Mol Cancer 19:105. 10.1186/s12943-020-01224-3 32522202 10.1186/s12943-020-01224-3 PMC7285594

[CR158] Zhang N, Gao Y, Yu S et al (2020b) Berberine attenuates Aβ42-induced neuronal damage through regulating circHDAC9/miR-142-5p axis in human neuronal cells. Life Sci 252:117637. 10.1016/j.lfs.2020.117637 32251633 10.1016/j.lfs.2020.117637

[CR162] Zhang Y, Zhao Y, Liu Y et al (2020c) Exploring the regulatory roles of circular RNAs in Alzheimer’s disease. Transl Neurodegener 9:35. 10.1186/s40035-020-00216-z 32951610 10.1186/s40035-020-00216-z PMC7504624

[CR155] Zhang F, Yao Y, Miao N et al (2022a) Neuroprotective effects of microRNA 124 in Parkinson’s disease mice. Arch Gerontol Geriatr 99:104588. 10.1016/j.archger.2021.104588 34906886 10.1016/j.archger.2021.104588

[CR163] Zhang Y-J, Zhu W-K, Qi F-Y, Che F-Y (2022b) CircHIPK3 promotes neuroinflammation through regulation of the miR-124-3p/STAT3/NLRP3 signaling pathway in Parkinson’s disease. Adv Clin Exp Med 32:315–329. 10.17219/acem/154658 10.17219/acem/154658 36306116

[CR154] Zhang F, Jiang J, Qian H et al (2023) Exosomal circRNA: emerging insights into cancer progression and clinical application potential. J Hematol OncolJ Hematol Oncol 16:67. 10.1186/s13045-023-01452-2 37365670 10.1186/s13045-023-01452-2 PMC10294326

[CR165] Zhao Y, Alexandrov P, Jaber V, Lukiw W (2016) Deficiency in the Ubiquitin Conjugating Enzyme UBE2A in Alzheimer’s Disease (AD) is Linked to Deficits in a Natural Circular miRNA-7 Sponge (circRNA; ciRS-7). Genes 7:116. 10.3390/genes7120116 27929395 10.3390/genes7120116 PMC5192492

[CR164] Zhao J, Lee EE, Kim J et al (2019) Transforming activity of an oncoprotein-encoding circular RNA from human papillomavirus. Nat Commun 10:2300. 10.1038/s41467-019-10246-5 31127091 10.1038/s41467-019-10246-5 PMC6534539

[CR166] Zheng Z-M (2019) Circular RNAs and RNase L in PKR activation and virus infection. Cell Biosci 9:43. 10.1186/s13578-019-0307-x 31149330 10.1186/s13578-019-0307-x PMC6537376

[CR168] Zhou L, Yang L, Li Y et al (2018a) MicroRNA-128 Protects Dopamine Neurons from Apoptosis and Upregulates the Expression of Excitatory Amino Acid Transporter 4 in Parkinson’s Disease by Binding to AXIN1. Cell Physiol Biochem 51:2275–2289. 10.1159/000495872 30537735 10.1159/000495872

[CR170] Zhou Z, Niu Y, Huang G et al (2018b) Silencing of circRNA.2837 Plays a Protective Role in Sciatic Nerve Injury by Sponging the miR-34 Family via Regulating Neuronal Autophagy. Mol Ther Nucleic Acids 12:718–729. 10.1016/j.omtn.2018.07.011 30098504 10.1016/j.omtn.2018.07.011 PMC6088565

[CR169] Zhou L-Y, Zhai M, Huang Y et al (2019) The circular RNA ACR attenuates myocardial ischemia/reperfusion injury by suppressing autophagy via modulation of the Pink1/ FAM65B pathway. Cell Death Differ 26:1299–1315. 10.1038/s41418-018-0206-4 30349076 10.1038/s41418-018-0206-4 PMC6748144

[CR167] Zhou J, Ye T, Yang Y et al (2024) Circular RNA vaccines against monkeypox virus provide potent protection against vaccinia virus infection in mice. Mol Ther 32:1779–1789. 10.1016/j.ymthe.2024.04.028 38659224 10.1016/j.ymthe.2024.04.028 PMC11184329

